# Genetic attenuation of ALDH1A1 increases metastatic potential and aggressiveness in colorectal cancer

**DOI:** 10.1002/1878-0261.70215

**Published:** 2026-02-03

**Authors:** Martina Poturnajova, Zuzana Kozovska, Ondrej Pos, Kristina Pavlov, Sachin Gulati, Peter Makovicky, Kristina Jakic, Monika Burikova, Eva Sedlackova, Barbora Svitkova, Silvia Tyciakova, Vojtech Bystry, Nicolas Blavet, Boris Tichy, Matej Hrnciar, Jaroslav Budis, Miroslav Tomas, Peter Dubovan, Georgina Kolnikova, Veronika Repaska, Nikoleta Mojzesova, Eva Zomborska, Daniel Pindak, Michal Mego, Tomas Szemes, Miroslava Matuskova

**Affiliations:** ^1^ Cancer Research Institute Biomedical Research Center of Slovak Academy of Sciences Bratislava Slovak Republic; ^2^ Comenius University Science Park Bratislava Slovak Republic; ^3^ Geneton Ltd. Bratislava Slovak Republic; ^4^ CEITEC – Central European Institute of Technology and Centre for Molecular Medicine Masaryk University Brno Czech Republic; ^5^ Faculty of Informatics and Information Technologies Slovak University of Technology in Bratislava Bratislava 4 Slovak Republic; ^6^ Department of Surgical Oncology National Cancer Institute Bratislava Slovak Republic; ^7^ Department of Surgical Oncology, Faculty of Medicine Slovak Medical University Bratislava Slovak Republic; ^8^ Department of Pathology National Cancer Institute Bratislava Slovak Republic; ^9^ Institute of Histology and Embryology, Faculty of Medicine Comenius University Bratislava Slovak Republic; ^10^ 2nd Department of Oncology National Cancer Institute Bratislava Slovak Republic; ^11^ Faculty of Medicine Comenius University Bratislava Slovak Republic

**Keywords:** aldehyde dehydrogenase, colorectal cancer, CRISPR‐Cas9, metastasis

## Abstract

Colorectal cancer ranks third in global incidence and second in cancer mortality. Patient‐derived models are irreplaceable for studying tumor biology. We established a human epithelial cell line from a rectal adenocarcinoma overexpressing cancer stem cell marker ALDH1A1, and we investigated the effect of ALDH1A1 knockout on tumor cell traits. The cell line and its CRISPR‐Cas9 ALDH1A1 knockouts were characterized by genomic and cytogenetic methods (CNV, WES, RNAseq, karyotype), *in vitro* (proliferation, response to chemotherapy, migration, invasion, apoptosis), and *in vivo* methods. We identified the landscape of somatic mutations and copy number alterations in the original tumor and the derived cell line. Genetic attenuation of ALDH1A1 was characterized by an increase in migratory potential and extensive metastatic ability, accompanied by reduced growth of subcutaneous xenografts and alterations in gene expression associated with inhibited proliferation and promoted invasion and metastasis, ultimately resulting in dysregulation of the Wnt signaling pathway. Increased metastatic potential was also confirmed in HT‐29 cells after ALDH1A1 genetic attenuation. CRISPR‐Cas9‐mediated editing led to functional, cellular, and molecular changes confirming the role of ALDH1A1 in colorectal cancer carcinogenesis.

## Graphical abstract

Aldehyde dehydrogenase 1A1 (ALDH1A1) is a cancer stem cell marker in several malignancies. We established a novel epithelial cell line from rectal adenocarcinoma with unique overexpression of this enzyme. Genetic attenuation of ALDH1A1 led to increased invasive capacity and metastatic potential, the inhibition of proliferation activity, and ultimately resulted in dysregulation of the Wnt signaling pathway.

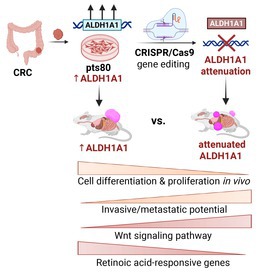

**Abbreviations**
ABCB1: ATP‐binding cassette subfamily B member 1
ADCY1: adenylate cyclase 1
AGTR1: angiotensin II receptor type 1
ALDH: aldehyde dehydrogenase
ANKS1B: Ankyrin repeat and sterile alpha motif domain containing 1B
BCAS1: brain enriched myelin‐associated protein 1
BME: basement membrane extract
CDH: cadherin
CEA: carcinoembryonic antigen
CK19, CK20: cytokeratin 19 and 20
CNV: copy number variations
COL6A3: collagen type VI alpha 3 chain
COPE: COPI coat complex subunit epsilon
CPNE6: copine 6
CPPED1: calcineurin‐like phosphoesterase domain containing 1
CRC: colorectal carcinoma
CRISPR‐Cas9: clustered regularly interspaced short palindromic repeat ‐ caspase 9
CSCs: cancer stem cells
CTH: cystathionine gamma‐lyase
CTNNBL1: catenin beta‐like 1
CYP26A1, CYP5A3: cytochrome P450 family members
DEG: differentially expressed genes
DHX37: DEAH‐Box helicase 37
DYNC1I1: dynein cytoplasmic 1 intermediate chain 1
EGFR: epidermal growth factor receptor
EIF3F: eukaryotic translation initiation factor 3 subunit F
ELDA: extreme limiting dilution analysis
EMT: epithelial–mesenchymal transition
EPCAM: epithelial cell adhesion molecule
EPHB3: EPH receptor B3
EYA4: eyes absent homolog 4
FCGR3A: Fc gamma receptor IIIa
FH: fumarate hydratase
FOLR1: folate receptor alpha
GNG11: guanine nucleotide‐binding protein subunit gamma 11
GRIP1: glutamate receptor interacting protein 1
IGF1: insulin like growth factor 1
JAK: Janus kinase
KLK13: kallikrein‐related peptidase 13
LARGE1: LARGE xylosyl‐ and glucuronyltransferase 1
LGR5: leucine rich repeat containing G protein‐coupled receptor 5
LINGO2: leucine rich repeat and Ig domain containing 2
LZTS3: leucine zipper tumor suppressor family member 3
MACC1: MET transcriptional regulator
MCOA4: nuclear receptor coactivator 4
MDGA2: MAM domain containing glycosylphosphatidylinositol anchor 2
MIB1: MIB E3 ubiquitin protein ligase 1
MLH1: MutL homolog 1
MSH2, MSH6: MutS homolog 2, 6
MSI: microsatellite instability
NBEA: neurobeachin
NCOA4: nuclear receptor coactivator 4
NEURL1B: neuralized E3 ubiquitin protein ligase 1B
NF‐κB: nuclear factor kappa B
NRXN2: neurexin 2
Oct4 (POU5F1): POU class 5 homeobox 1
PCDH: protocadherin
PCSK9: proprotein convertase subtilisin/kexin type 9
PDCD4: programmed cell death 4
PECAM1: platelet and endothelial cell adhesion molecule 1
PI3K: phosphatidylinositol−3‐kinase
PKD1L2: polycystin‐1 L2
PLAAT4: phospholipase A and acyltransferase 4
PMS2: PMS1 homolog 2, mismatch repair system component
RA: retinoic acid
RARRES: retinoic acid receptor responder protein 1
RARα, RARb: retinoic acid receptor alpha, beta
RBFOX1: RNA binding fox‐1 homolog 1
ROS: reactive oxygen species
SLC16A14, SLC2A13, SLC30A10, SLC34A2: solute carrier family members
SMOX: spermine oxidase
SNAI2: snail family transcriptional repressor 2
Sox2: SRY‐Box transcription factor 2
SPAG9: sperm‐associated antigen 9
SPATA13: spermatogenesis associated 13
ST6GALNAC6: ST6 N‐Acetylgalactosaminide alpha‐2,6‐sialyltransferase 6
STAT: signal transducer and activator of transcription
STC: solute carrier family member
STRA6: signaling receptor and transporter of retinol
STRAP: serine/threonine kinase receptor associated protein
STX4: syntaxin
STXBP5L: syntaxin binding protein 5 L
TBXAS1: thromboxane A synthase 1
TCERG1L: transcription elongation regulator 1 like
TENM3: teneurin transmembrane protein 3
TGF‐β: transforming growth factor beta
TGM2: transglutaminase 2
THSD7B: thrombospondin type 1 domain containing 7B
TICs: tumor‐initiating cells
TMTC1: transmembrane O‐mannosyltransferase targeting cadherins 1
TNFSF15: TNF superfamily member 15
TTN: titin
Twist1: twist family BHLH transcription factor 1
UBE2D4: ubiquitin conjugating enzyme E2 D4
URGCP: upregulator of cell proliferation
VEGF: vascular endothelial growth factor
VIM: vimentin
WES: whole exome sequencing
XPO4: exportin 4
ZDHHC17: Zinc finger DHHC‐type palmitoyltransferase 17
ZEB2: Zinc finger E‐box binding homeobox 2
ZFP: Zinc finger protein

## 1 Introduction

Colorectal cancer (CRC) is one of the most frequently diagnosed cancers worldwide and the cause of the death of 930 000 patients annually [1]. There is an urgent need for further research using novel preclinical models derived from patient cancer tissue, which accurately represent and capture the properties of the original tumor. Establishing new, patient‐derived 2D and 3D cell models provides a prerequisite for research on tumor properties such as stemness, chemoresistance, and metastasis.

The aldehyde dehydrogenase (ALDH) family consists of 19 isoforms involved in the metabolism of aldehydes of endogenous and exogenous origin. Each ALDH isoform has a specific expression pattern; some have prognostic value in cancer [2]. Higher ALDH activity was discovered in cancer stem cells (CSCs) and linked to their stemness. Moreover, ALDH expression is tissue‐ and tumor‐specific [3].

The ALDH1A1 facilitates the oxidation of retinaldehyde to retinoic acid, the oxidation of acetaldehyde, and lipid peroxidation‐derived aldehyde. It is an essential cytosolic enzyme involved in various cellular processes, including cell survival and protection against oxidative damage. It detoxifies harmful aldehydes and reactive oxygen species (ROS) and affects drug metabolism [4, 5]. Besides the ALDH1A3, it converts vitamin A to its active metabolite, retinoic acid. It is responsible for retinoic acid‐mediated signaling and cell differentiation [4, 5].

The ALDH1A1 is a CSC marker in breast, lung, colon, and stomach carcinoma, with none or low levels in corresponding normal tissues [6]. Its overexpression in tumor cells correlates with a poor prognosis and is associated with increased proliferation, therapy resistance, motility, and tumorigenicity. Intervention into ALDH1A1 production could affect these essential characteristics, including stemness and cellular differentiation.

We have previously shown that the decrease of ALDH1A1 expression in HT‐29 cells is linked to the alterations in response to chemotherapy and tumorigenic potential [7]. Acquired chemoresistance was accompanied by decreased ALDH1A1 and increased ALDH1A3 expression. These changes were associated with a reduced ability to form subcutaneous xenografts and a significantly increased metastatic potential [8].

CRISPR‐Cas9 genome editing has been utilized previously in CRC research to construct preclinical models, prepare genome‐wide screening libraries, and investigate non‐coding RNAs, genes associated with drug resistance, hereditary CRC, or immuno‐ and gene therapy [9]. However, this technique has not been used previously to attenuate the ALDH1A1 stem cell marker.

We aimed to investigate how endogenous ALDH1A1 overexpression in a patient‐derived cell line is associated with biological characteristics and expression profile. We employed the CRISPR‐Cas9 approach to stably attenuate the *ALDH1A1* gene and analyzed its impact using genomics, transcriptomics, and *in vitro* and *in vivo* methods.

## 2 Material and methods

### 2.1 Characteristics of the patient and processing of tumor tissue

The tumor specimen was obtained from an 82‐year‐old European male with histologically confirmed moderately differentiated tubular adenocarcinoma of the rectum. The patient's written informed consent was obtained before collecting the tumor tissue (in July 2019) and peripheral blood (in May 2023) at the National Cancer Institute, Bratislava. The procedure was approved by the National Cancer Institute Ethics Committee, Bratislava, Slovakia (study number GIT01). All study methodologies performed on pts80 human samples were undertaken according to ethical principles for medical research set by the Declaration of Helsinki. The patient's tumor tissue was verified by a pathologist and processed: one‐third was fixed with 4% paraformaldehyde and subjected to histological and immunohistochemical examination; one‐third was immediately frozen and stored at −80 °C for biobanking, later used for RNA and genomic DNA (gDNA) isolation. The vital tissue was transferred in a transport medium composed of DMEM/Ham's F12 medium (PAN Biotech, Aidenbach, Germany), supplemented with 10% FBS (PAN Biotech), and 100 μg·mL^−1^ gentamicin (Sandoz, Basel, Switzerland), 5 μg·mL^−1^ amphotericin B, 100 μg·mL^−1^ penicillin, 50 μg·mL^−1^ streptomycin, 20 μg·mL^−1^ metronidazole and transferred ice‐cold immediately to further processing.

### 2.2 Establishment of patient‐derived cell line and cell culture

The tumor tissue was repeatedly washed in the transport medium, cut into small pieces, and then mechanically and enzymatically processed using the Tumor Dissociation Kit (Miltenyi Biotec, Gladbach, Germany). Adherent cultures were established by plating 1.9 × 10^5^ cells per cm^2^ of Petri dish and cultivated in DMEM/Ham's F12 medium supplemented with 10% FBS and 10 μg·mL^−1^ gentamicin (Sopharma, Sofia, Bulgaria).

HT‐29 (ATCC® HTB‐38TM) (RRID: CVCL_0320) and HCT 116 (ATCC® CCL‐247) (RRID: CVCL_0291) were purchased from ATCC®. Cells were cultured in high‐glucose DMEM with 10% FBS and 10 μg·mL^−1^ gentamicin, maintained at 37 °C in a humidified atmosphere and 5% CO_2_, and were tested every month for mycoplasma contamination by PCR. All experiments were performed using mycoplasma‐free cells. Cell lines were authenticated in the past 3 years using STR profiling by Generi Biotech Ltd. (Hradec Kralove, Czech Republic).

### 2.3 Immunophenotyping

Cells were detached from the Petri dish with the Accutase (PAA Laboratories (Pasching, Austria), Thermo Scientific (Waltham, MA, USA)). Two hundred and fifty thousand cells per sample were washed with PBS and pelleted. Cells were resuspended in 50 μL of PBS with 1% BSA. Primary antibodies in concentrations according to the manufacturer's instructions were added: CD326‐PE (EpCAM, #130‐093‐440, Miltenyi Biotec), CD324‐APC (E‐cadherin, #130‐111‐993, Miltenyi Biotec), CD44‐APC (#130‐113‐331, Miltenyi Biotec), CD44v6‐PE (FAB3660P, R&D Systems, Abingdon, UK), cMET‐APC (FAB3582A, R&D Systems), CD325‐APC (N‐cadherin, #130‐116‐274, Miltenyi Biotec), CD87‐APC (#130‐114‐893, Miltenyi Biotec), CD338‐PE (ABCG2, #12888841, eBiosciences, Altrincham, UK); and incubated for 15 min in the dark. Relevant fluorochrome‐conjugated isotypes were used as controls. Cells were resuspended in 200 μL of PBS with 0.1 μL·mL^−1^ DAPI and analyzed using a BD FACSCanto™ II flow cytometer equipped with the FACSDiva program (Becton Dickinson, Franklin Lakes, NJ, USA). The obtained data were evaluated in the fbs express program (*De Novo* Software, Pasadena, CA, USA).

### 2.4 Cytogenetic analysis

Karyotyping of cells was performed in the exponential growth phase using a standard air‐drying method. In brief, after incubation with 0.1 μg·mL^−1^ colchicine for 4 h, cells were treated with hypotonic solution (0.075 m KCl) for 15 min at 37 °C and then fixed in Carnoy's fixative (methanol: acetic acid; 3 : 1). Fixed cells were dropped from a distance of about 50 cm onto dry slides, air dried, and stained with 4% Giemsa stain. A total of 100 metaphases were automatically detected and further analyzed for chromosomal aberrations in a double‐blinded manner using the Zeiss Axio Imager Z2 fluorescence microscope (Carl Zeiss, Oberkochen, Germany) and the Metafer 3.6 software (MetaSystems, Altlussheim, Germany). The selection of metaphase and criteria for structural aberrations complied with the generally accepted recommendations [10]. Structural aberrations, including chromatid gaps and breaks, isochromatid gaps and breaks (acentric), and exchanges (dicentrics, rings), were analyzed manually.

### 2.5 Whole exome sequencing

Whole exome PCR‐free sequencing (WES) libraries were prepared utilizing xGen™ DNA Library Prep EZ UNI (IDT), using 500 ng of DNA as input. After index adapter ligation, libraries were quantified using the KAPA Library Quantification Kit (Roche, Basel, Switzerland). The final library pool was sequenced with the MGI sequencer DNBSEQ‐G400 in paired‐end 2 × 150 mode.

### 2.6 Data processing and variant calling

The raw sequencing data were preprocessed to remove adapter sequences and trim low‐quality bases. The cleaned reads were aligned to the human reference genome GRCh38 using BWA alignment software [11]. Duplicate reads were identified and removed using the UMI‐aware version of MarkDuplicates from Picard Tools [12]. Somatic small variants, including single‐nucleotide variants (SNVs) and small insertions and deletions (indels), were identified from tumor and corresponding normal tissue samples using the SomaticSeq variant caller [13], a meta‐caller that aggregates calls from multiple tools, including strelka2 [14], vardict [15], mutect2 [16], somaticsniper [17], lofreq [18], muse [19], and varscan2 [20].

### 2.7 SNVs and indels variant annotation

Identified variants were annotated using Ensembl's Variant Effect Predictor (VEP) tool [21], utilizing its full annotation cache. Pathogenicity scores from the Evolutionary Model of Variant Effect (EVE) [22], Combined Annotation‐Dependent Depletion (CADD) [23], and PolyPhen‐2 [24] were annotated, alongside cancer‐specific annotations from clinical databases such as fOne, MD Anderson, TruSight Oncology, and the Cancer Gene Census (CGC).

### 2.8 CNV detection

Mapped raw sequencing reads were counted in 500 kb bin intervals, representing a minimal size limit of copy number variations (CNV) detection. These bin counts underwent normalization based on sample size and GC content to address biases and variations. For the identification of CNVs on autosomes, we combined the results of the control‐freec tool [25], cnvkit [26], and an in‐house algorithm using the normalized binned read counts, incorporating a negative binomial distribution for individual bin counts to handle overdispersion, followed by using a dynamic Bayesian network model for holistic CNV predictions. The results were combined for consensus CNV calls, including bins predicted by at least two callers.

### 2.9 CNV pathogenicity prediction

For the clinical significance prediction of CNV findings, we have utilized an automated ISV method [27]. This machine learning‐based approach requires the start‐end genomic coordinates of a structural variant on a particular chromosome and the type of change (i.e., loss or gain of the genomic region) but does not consider the exact copy number to predict its pathogenicity. We have used a conservative model to classify CNV into three categories: benign, uncertain significance, and pathogenic. The threshold values of the ISV score were set as follows: 0.00–0.05 benign; 0.05–0.95 uncertain; 0.95–1.00 pathogenic. Semi‐automated classification using the generally accepted ACMG/ClinGen standards [28] was not included since it needs to rely on other tools or manual classification by experts.

### 2.10 RNA sequencing

Total RNA was isolated using Trizol (Thermo Fisher Scientific) according to the manufacturer's protocol. If the A260nm/A230nm ratio was lower than 1.8, the RNA sample was purified by ethanol precipitation. RNA integrity was checked on the Fragment Analyzer using RNA Kit 15 nt (Agilent Technologies, Santa Clara, CA, USA). 500 ng of total RNA was used as input for library preparation using QuantSeq 3′ mRNA‐Seq FWD with UDI 12 nt Kit (v.2) (Lexogen, Vienna, Austria) in combination with UMI Second Strand Synthesis Module for QuantSeq FWD (Lexogen). Quality control for library quantity and size distribution was performed using the QuantiFluor dsDNA System (Promega, Madison, WI, USA) and the High Sensitivity NGS Fragment Analysis Kit (Agilent Technologies). The final library pool was sequenced using Illumina technology in single‐end mode, yielding 10–15 million reads per sample.

### 2.11 Bioinformatic processing of RNA sequencing data

Raw reads were checked for quality (FastQC, MultiQC, minion, swan), preprocessed (Trimmomatic, FastQC, MultiQC), and mapped (STAR, Samtools, MultiQC) to the reference genome GRCh38‐p10 with gene annotation. Only uniquely mapped and assigned reads were counted with featureCounts. The library preparation kit was forward strand‐specific, and only reads in the forward strand were considered.

To estimate the overall sample quality, rRNA content estimate (fastq_screen), Read duplication rate (dupRadar, Picard tools), Sequenced (targeted) regions (RSeQC, Picard tools), 5′/3′ coverage bias (Picard tools), Expressed gene biotypes (featureCounts) were performed. A predefined cut‐off value was set to *P*‐value < 0.05 and logFC > 1 (FC = fold change). Differential gene expression was calculated by DESeq2.

Gene ontology was performed using the PANTHER database version 18.0 [29]. Gene set enrichment analysis and signaling pathways were generated using clusterprofiler version 4.10.0 [30], enrichplot version 1.22.0 [31], and dose version 3.28.1 [32] r packages, KEGG database version 109.1 [33], and Reactome database version 85 [34].

### 2.12 Cell proliferation assay and doubling time assessment

Cells (3 × 10^3^ cells per well) were plated in hexaplicates in 96‐well plates, and their proliferation, based on cell confluence analysis, was monitored for 6 days by IncuCyte ZOOM™ Kinetic Imaging System (Essen BioScience) and analyzed by IncuCyte ZOOM™ software. Values were expressed as means ± SD (*n* = 6). To determine doubling time, the plot of cell confluence (*y*‐axis) versus time (*x*‐axis) in hours was used. The *y*‐axis was converted to a logarithmic scale, and the plot section with linear dependence (means exponential growth) was taken. The trendline of *x* and *y* values from the section on exponential growth gives *y* = A.e^Bx^. Doubling time was set as LN 2/B = 0.693/B.

### 2.13 Cell viability assay

Tumor cells (3 × 10^3^ per well) were plated in 96‐well white plates (Greiner) in culture medium and allowed to adhere overnight. The next day, treatment with the gradient of 5‐FU (Sigma‐Aldrich, Darmstadt, Germany; 0.125–2 μg·mL^−1^) and oxaliplatin (Sigma‐Aldrich; 0.125–4 μg·mL^−1^) started. Cells were incubated for 6 days, and cytotoxicity was assessed using the CellTiter‐Glo Luminescent Cell Viability Assay (Promega) according to the manufacturer's instructions. The relative luminescence was measured on a GloMax Discover Microplate Reader (Promega) and expressed as mean ± SD (*n* = 6) with untreated cells as a reference. The IC_50_ values were calculated using the calcusyn software (Grimsby, England).

### 2.14 Migration assay

Sixty‐five thousand cells per well were plated in 10 replicates on ImageLock 96‐well Plates (Sartorius, Göttingen, Germany) and allowed to adhere for 24 h. Confluent monolayers were wounded with the 96‐well WoundMaker™ (Essen BioScience) and washed. Then, DMEM containing 3% FBS was added, and images were taken every 3 h using the IncuCyte ZOOM Kinetic Imaging System for the next 2 days. Cell migration was evaluated using IncuCyte ZOOM™ software based on the relative wound density measurements. The cell confluence in the wound was expressed as mean ± SD (*n* = 10).

### 2.15 F‐actin staining

Fifteen thousand cells were seeded on the glass coverslips in a 48‐well plate (Greiner). The cells proliferated for 2 days and then were washed with PBS. They were fixed with 4% paraformaldehyde at room temperature (RT) for 20 min, washed with PBS, and then permeabilized with 0.05% Triton X‐100 in PBS at RT for 15 min. Non‐specific binding was blocked with 3% BSA in PBS at 37 °C for 30 min. After incubation (1 h at 37 °C in the dark) with Alexa Fluor 488 Phalloidin (Thermo Fisher Scientific), the cells were washed with PBS, and the nuclei were counterstained with DAPI. The cells were mounted on slides with a mounting medium (Sigma‐Aldrich). Actin fibers were visualized with a fluorescent microscope (Axio Imager, Zeiss, Oberkochen, Germany) using ISIS software (MetaSystems GmbH) at 630× magnification.

### 2.16 Detection of apoptosis by Annexin V assay

The 15 000 cells were seeded in triplicate on 24‐well plates and incubated for 72 h. Cells were detached by Accutase, washed with Annexin V binding buffer, and incubated for 20 min with APC‐conjugated Annexin V (Thermo Fisher Scientific) according to the manufacturer's recommendations. Propidium iodide (2 μg·mL^−1^) was used for the detection of necrotic cells. Analysis was performed using a BD FACS Canto II flow cytometer equipped with the FACS Diva program, and data were analyzed with the FCS Express program. Values represent means ± SD (*n* = 3).

### 2.17 Colony‐forming assay in soft agar

Six‐well plates covered with culture medium containing 20% FBS and 0.6% low‐melting‐point (LMP) agarose (Sigma) were used after polymerization to plate 2 × 10^3^ and 4 × 10^3^ cells resuspended in culture medium containing 20% FBS and 0.6% LMP agarose. The cells were allowed to grow and form colonies for 14 days, the colonies were counted manually, and the pictures of the colonies were taken. Only colonies with more than 30 cells were included in the analysis. Clonogenic abilities were calculated as a ratio of proliferating colonies and the number of plated cells in x 100. Values were expressed as median (*n* = 12 for HT‐29 and *n* = 16 for pts80) with min/max, and statistical significance was calculated by Mann–Whitney test.

### 2.18 Colonosphere‐forming assay

Serial dilutions of parental or engineered cells were prepared in DMEM/F12 (PAN Biotech) medium supplemented with B27 (Miltenyi Biotec), 20 ng·mL^−1^ basic fibroblast growth factor (bFGF; Miltenyi Biotec), 50 ng·mL^−1^ epidermal growth factor (EGF; Miltenyi Biotec), 2 mm GlutaMAX (Gibco, Waltham, MA, USA), and 10 μg·mL^−1^ gentamicin. Cells were seeded into ultra‐low attachment (ULA) 24‐well flat‐bottom plates (Corning, NY, USA). The formation of colonospheres was examined by light microscopy 9 days later. The viability of spheroids was evaluated by CellTiter‐Glo® 3D Cell Viability Assay (Promega, Madison, WI, USA) according to the protocol and measured on a GloMax Discover Microplate Reader (Promega). Values were expressed as the median of triplicates ± SD.

### 2.19 Histology and immunohistochemistry

Tissue specimens were fixed in 10% neutral formaldehyde (Sigma‐Aldrich) for 24 h, embedded in paraffin blocks, and cut on a Hyrax M40 rotary microtome (Zeiss). The tissue sections were placed on Star Frost® slides (Knittel Glass, Braunschweig, Germany) and stained with hematoxylin–eosin (Bamed, Ceske Budejovice, Czech Republic). For immunohistochemistry, heat‐induced antigen retrieval was performed by incubating the samples for 20 min in a PT Link (Dako, Santa Clara, CA, USA) at 96 °C using EnVision Flex Target Retrieval Solution high‐pH (Dako). Then, the slices were cooled down to room temperature and washed for 5 min in a wash buffer (EnVision Flex Wash Buffer, Dako) prior to loading onto the automated DAKO Autostainer Link 48. Endogenous peroxidase was blocked by 5 min incubation with FLEX peroxidase Block (DAKO). Sections were then incubated with primary antibodies: Carcinoembryonic Antigen clone II‐7, Cytokeratin 7 clone OV‐TL 12/30, Cytokeratin 19 clone RCK108, Cytokeratin 20 clone KS 208, Ki‐67 antigen clone MIB‐1, and Vimentin clone V9 for 20 min at RT (FLEX, DAKO), followed by incubation with LSAB2 System‐HRP, Biotinylated Link for 15 min, and then Streptavidin‐HRP for 15 min. Positive staining was visualized by 3.3′‐Diaminobenzidine (DAB substrate‐chromogen solution, DAKO) after 5 min; counterstaining was performed with hematoxylin (FLEX, DAKO) for 5 min. Slides were mounted with paramount aqueous mounting media (DAKO), and the staining patterns were analyzed using an Olympus microscope BX46.

### 2.20 CRISPR‐Cas9 genome editing

We designed a target sequence for guide RNA (gRNA) by the Rational design of the CRISPR/Cas target online tool [35] to identify possible targets based on the presence of the PAM motif immediately downstream of the 3′ end of our target sequence. The software identified 20 bp sequences before PAM motifs within *ALDH1A1*; we selected unique target sequences in different parts (ORF and promoter) of the *ALDH1A1* gene (NM000689.4) to lower the probability of off‐targets and cut the central part of the gene. For specificity check, the human genome GRCh37/hg19 was used. For oligo design, the cohesive ends were added to create BsmBI restriction sites. One guanine was added upstream of the 5′ end of the 5′ → 3′ strand to enhance expression (Table 1). The insert was cloned into the LentiCRISPR v2 vector (#52961, Addgene, Watertown, MA, USA) by *BsmBI* restriction sites according to the Addgene protocol and confirmed by sequencing.

**Table 1:** Oligos specific for the ALDH1A1 gene used for ligation into the LentiCRISPRv2 vector.

Label	Oligo sequences 5′ → 3′	
gRNA1	CACCG TTTGCATACTCGGATACGAT	CAAACGTATGAGCCTATGCTA CAAA
gRNA2	CACCG GAATCTTCAAATCGGTGAGT	CCTTAGAAGTTTAGCCACTCA CAAA
gRNA3	CACCG AGCATCCATAGTACGCCACG	CTCGTAGGTATCATGCGGTGC CAAA

To produce lentiviral particles, we used lentiviral‐based transfection of HEK293T cells using the CalPhos™ Mammalian Transfection Kit (Takara). Briefly, 2.5 × 10^6^ HEK 293 T cells were seeded into a 10 cm Petri dish and transfected with 6 μg pCMV‐VSV‐G (#8454, Addgene), 6 μg psPAX2 (#12260, Addgene), 10 μg expression vector pLentiCRISPRv2/gRNA for ALDH1A1 in the presence of 0.25 m CaCl_2_ and 2xHBS solution. Transfected packaging cells were incubated at 37 °C overnight. The medium was replaced, and HEK293T cells were then maintained for the next 72 h to produce lentiviral particles. The lentivirus‐containing medium was filtered through a 45 μm filter, and the virus was precipitated with PEGit™ (SBI) at 4 °C overnight. The precipitate was then centrifuged at 1500 ***g*** for 30 min at 4 °C, resuspended in cold PBS, aliquoted, and stored at −80 °C until use.

For the preparation of *ALDH1A1* knockouts, 25 μL of the virus was added dropwise to 10^5^ pts80 or HT‐29 cells in one well of a 6‐well plate in medium with 10 μg·mL^−1^ of polybrene. To enhance the efficiency of cleavage, we employed a combination of two lentiviruses with distinct guide RNAs. The same protocol was used for the preparation of control cells (pts80 EV ctrl) containing the LentiCRISPR v2 vector with no gRNA sequence (EV = empty vector). Transduced cells were selected with 0.8 μg·mL^−1^ (pts80) and 0.75 μg·mL^−1^ (HT‐29) puromycin for 7 days. Transduced colonies of sufficient size were trypsinized and transferred to a 96‐well plate to obtain one‐cell‐derived colonies for further propagation.

### 2.21 Verification of gene editing in genomic DNA by PCR and sequencing

Genomic DNA from transduced cells was isolated by DNA‐zol (DN 127, Molecular Research Center). Fifty micrograms of genomic DNA was used for PCR. The primers (Table 2) were used according to the localization of the gRNA's sequence. The PCR products were sequenced to confirm the successful editing of the *ALDH1A1* gene.

**Table 2:** List of primers used for PCR to confirm the gene editing.

Label	Forward primer sequence 5′ → 3′	Reverse primer sequence 5′ → 3′	Product length
*ALDH1A1 1, 2*	GGTGTTACAAATAAGTAGTGTCGTT	GACCTGCATTTTGCATGCCT	385 bp
*ALDH1A1 3*	CTGCTTATGAGGCACTACAGTCTAT	TTTGAGAGCTCTAGCTACCCATCC	284 bp

### 2.22 Gene expression analysis

Total RNA was isolated from 1 × 10^6^ cells using Trizol® according to the manufacturer's instructions. RNA was depleted from gDNA using the RapidOut DNA Removal kit (Thermo Fisher Scientific), and 5 μg of RNA was reverse‐transcribed using the RevertAid H minus First Strand cDNA Synthesis Kit (Thermo Fisher Scientific) according to the protocol. Quantitative RT‐qPCR was performed in a Bio‐Rad CFX96™ Real‐Time PCR Detection System (Bio‐Rad, Hercules, CA, USA). The PCR consists of 1 μL of cDNA, primers (Table S1) (f.c. 0.3 pmol·μL^−1^), and AmpliTune® qPCR EvaGreen® Mix (Selecta Biotech, Bratislava, Slovakia) according to the manufacturer's instructions. The *HPRT1* and *COPE* expressions were set as endogenous reference controls. Gene expression was calculated as normalized fold change expression using the $2^{-\Delta \Delta C_{q}}$ method by CFX Manager™ (Hercules, CA, USA) (Version 1.5). Data were expressed as means ± SD (*n* = 4).

### 2.23 Evaluation of ALDH activity

ALDH activity was determined by ALDEFLUOR™ assay (Stem Cell Technologies, Vancouver, Canada) according to the manufacturer's protocol. ALDH1A1‐selective inhibitor NCT‐501 (Sigma‐Aldrich) was used for inhibition of ALDH1A1 activity [36]. Samples containing 0.5 × 10^6^ cells were treated with 15 μmoL·L^−1^ DEAB (Stem Cell Technologies) or 80 μmoL·L^−1^ NCT‐501 for 45 min at 37 °C in the dark. Analysis was performed on a CytoFLEX S flow cytometer (Beckman Coulter, Brea, CA, USA), and data were analyzed with the Kaluza Analysis software version 2.2.1 (Beckman Coulter).

### 2.24 *In vivo* studies

The *in vivo* study was approved by the Institutional Ethics Committee (EK/Zv‐10/2017, EK/Zv‐07/2019) and the State Veterinary and Food Administration of the Slovak Republic under approvals No. Ro‐207/18‐221/3 and Ro‐290‐3/2020‐220. Six‐ to 8‐week‐old SCID/bg mice (CD17 Cg Prkdscid Lystbg/Crl, Charles River) were used under institutional guidelines and approved protocols. Mice were bred under aseptic conditions in individually ventilated cages (NexGen Allentown, NJ, USA). Before tumor cells administration, animals (both sexes) were randomly divided into groups. The 2 × 10^6^ pts80 cells in 50 μL 1 : 1 ECM/DMEM mixture were bilaterally administered subcutaneously into the flanks. Four or five mice per group were used. The 1 × 10^5^ HT‐29 cells in 100 μL DMEM were bilaterally administered subcutaneously into the flanks. Xenografts were measured by caliper three times a week, and volume was calculated according to the formula: V = 0.5236 × ((width + length)/2)^3^. Because the pts80 KO and HT‐29 KO xenografts grew at different rates, animals were euthanized when xenografts reached the approved endpoint size of 1 cm^3^. Xenografts were excised and weighed, and mice were checked for visible metastasis. Xenografts, lungs, liver, spleen, and colon were subjected to molecular analysis and histological examination.

For extreme limiting dilution analysis (ELDA), 10 times gradually decreasing number of parental pts80 and 1C3 KO cells with starting number of 1 million to 100 cells per dose were injected subcutaneously in the flank of SCID/beige mice in 50 μL of serum‐free medium (ratio 1 : 1 with ECM), 3 applications to one mouse. Similarly, for HT‐29 parental cells and G5‐3 ALDH1A1 knockout, limiting dilutions starting from 100 000 cells to 100 cells were applied s.c. in 50 μL of serum‐free medium. The animals were inspected twice a week for the tumor incidence and considered tumor‐free when no palpable rigid structure exceeding 1 mm^3^ could be detected. Growing xenografts were measured, and volumes were calculated as stated above. Animals were sacrificed when the tumors exceeded 1 cm^3^ in accordance with the ethical guidelines or at the experiment endpoint. Mice were designated tumor‐free at the experiment endpoint, when no tumor growth was detectable at necropsy. Xenograft penetrance was determined as the proportion of growing tumors among all inoculations at a given cell number. The frequency of tumor‐initiating cells was determined as described in [37]. Tumor take rates for every group and cell number at endpoint were uploaded to ELDA software [38] and stem cell frequency and confidence intervals were estimated.

### 2.25 Detection of micrometastases

After the mechanical homogenization of tissue samples, the gDNA was extracted by innuPREP DNA Mini Kit 2.0 (IST Innuscreen, Berlin, Germany) according to the manufacturer's protocol. From each sample, 200 ng of gDNA was subjected to duplex qPCR analysis in GoTaq® Probe Master Mix (Promega) for the presence of human β‐globin gene and mouse Rapsyn gene (f.c. 0.1 pmol·μL^−1^) using dual‐labeled probes FAM‐BHQ1‐β‐globin and JOE‐BHQ1‐Rapsyn (both in f.c. 0.267 pmol·μL^−1^). The primers and probes used are listed in Table S1. PCR was performed using the Aria MX Real‐Time PCR System (Agilent Technologies). Genomic DNA isolated from PBMC of the pts80 patient's blood was used as a positive control for β‐globin and gDNA from mouse healthy lung tissue as a positive control for Rapsyn. The analysis was performed by absolute qPCR quantification of β‐globin and Rapsyn signals using 5‐point 5‐fold serial standard curves. The amount of human gDNA of pts80 metastatic cells in mouse tissue was calculated according to the β‐globin standard curve. The PCR was optimized to achieve sufficient efficiency, and values were expressed as means ± SD (*n* = 3).

### 2.26 Statistical analysis

graphpad prism (Boston, MA, USA) (version 6) was used for statistical analysis. The normality of distribution was tested using the Shapiro–Wilk test. Depending on the normality of the data distribution, the Student's unpaired t‐test or the Mann–Whitney test was applied for hypothesis testing to compare the means of the two groups. Differences between more than two groups were tested using one‐way analysis of variance (ANOVA) or the Kruskal—Wallis *H* test, followed by multiple comparisons. The growth dynamics of xenografts were statistically evaluated by a paired non‐parametric Wilcoxon test. Differences with *P* < 0.05 were considered statistically significant (*P < 0.05, **P < 0.01, ***P < 0.001). All analyses were performed with technical replicates (N specified in every method).

## 3 Results

### 3.1 Clinicopathological data

**Fig. 1:**
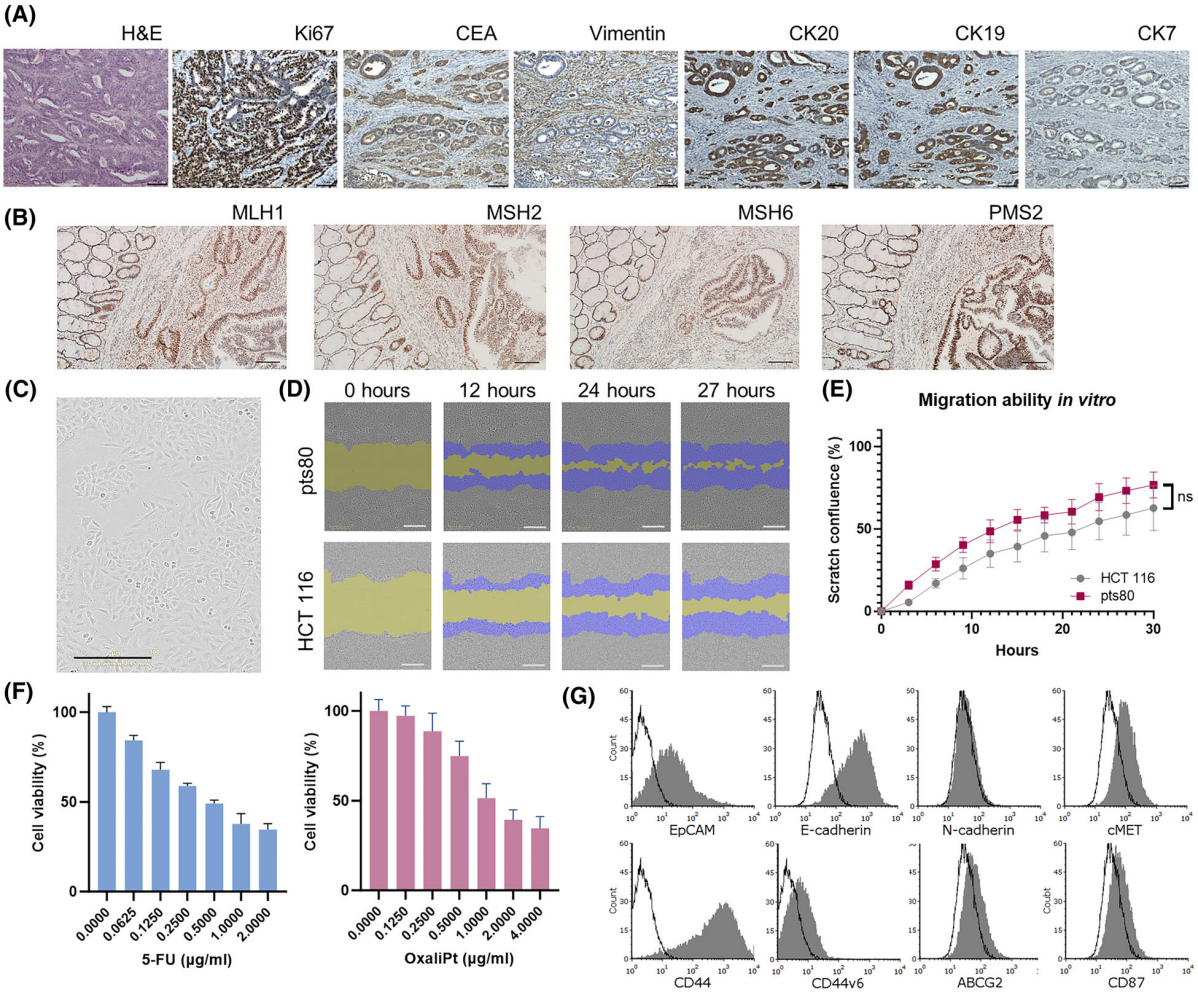
Characterization of patient's tumor tissue and established pts80 cell line: biological properties and markers. (A) Hematoxylin–eosin staining (H&E) and immunohistochemistry for Ki67, CEA, Vimentin, and cytokeratins 20, 19, 7 (CK20, CK19, CK7) in the patient's tumor tissue, magnification 100×, Olympus microscope BX46, representative pictures of triplicates, scale bar: 100 μm. (B) Immunohistochemistry for mismatch repair proteins MLH1, MSH2, MSH6, PMS2 in tumor tissue, magnification 40×, scale bar: 100 μm Olympus microscope BX46, representative pictures of triplicates. (C) Epithelial pts80 cell line growing in adherent conditions, magnification 10×, scale bar: 300 μm, Incucyte**®** Zoom System representative picture after cell line stabilization. (D, E) The migration ability of pts80 cells compared to HCT 116 cell line; wound healing assay; analyzed by Mann–Whitney test (*n* = 10), representative results of three independent experiments, mean ± SD, scale bar: 300 μm, Incucyte**®** Zoom System. (F) Chemosensitivity to 5‐fluorouracil (5‐FU) and oxaliplatin (OxaliPt) analyzed by Mann–Whitney test (*n* = 6), results of three independent experiments, mean ± SD. (G) Cell surface markers analyzed by flow cytometry, representative results of three independent experiments (ns – not significant, *P* ≥ 0.05).

The tissue specimen was obtained from a histologically verified moderately differentiated tubular adenocarcinoma of the rectum, Grade 2, pT3pN0pMX, from an 82‐year‐old European male. The patient was not subjected to neoadjuvant chemotherapy before surgery. Histological examination confirmed no sign of metastatic dissemination in blood vessels or lymph nodes (14 lymph nodes examined). Lynch syndrome and sporadic MSI due to his age were unlikely. The diagnosis of rectal adenocarcinoma was supported by the presence of carcinoembryonic antigen (CEA) and positivity for cytokeratins 19 and 20 (CK19, CK20). Malignant cells displayed a high proliferative activity (80% of cells showed Ki67 nuclear positivity) and negativity for Vimentin (Fig. 1A). Immunohistochemistry also showed a positive intact nuclear expression for mismatch repair proteins MLH1, MSH2, MSH6, and PMS2 (Fig. 1B) supporting the diagnosis of sporadic adenocarcinoma.

### 3.2 Establishment and characterization of a novel colorectal cancer cell line

We established an adherent cell line from the vital tumor tissue and denoted it as pts80. Unlike most of the primary cell cultures, the pts80 culture did not contain cancer‐associated fibroblasts. Cells displayed epithelial morphology in adhesion culture (Fig. 1C) and grew with a doubling time of 18.6 h. We have also administered the single‐cell suspension prepared from patients' cancer tissue directly subcutaneously to the flank of SCID/bg mice. Still, after the second *in vivo* passage, the xenograft did not grow.

The cell line exhibited migration ability. The migratory potential was similar to the colorectal cancer‐derived cells HCT 116 (Fig. 1D,E). Oxaliplatin and 5‐fluorouracil (5‐FU) are chemotherapeutics commonly used in the treatment of CRC; the sensitivity of the pts80 cell line was determined. The cells were found to be sensitive to both 5‐FU and oxaliplatin (Fig. 1F), with 50% inhibitory concentrations (IC50) of 0.49–0.54 μg·mL^−1^ for 5‐FU and 1.08–1.54 μg·mL^−1^ for oxaliplatin.

Examination of surface markers confirmed that the pts80 cell line has characteristics of epithelial cells (79% positivity for EpCAM and 93% positivity for E‐Cadherin) and lacks the characteristics of cells of mesenchymal origin (negativity for N‐cadherin). We observed 99% positivity for the CD44, and 30% of the cells expressed the CD44 variant 6 (CD44v6), which is strongly associated with CRC metastasis [39]. Above 70% of the population was positive [36] for receptor tyrosine kinase c‐MET, considered another marker of tumor aggressiveness and invasiveness. One‐third of the cells were positive for the ABCG2 transporter and the urokinase‐type plasminogen activator receptor (uPAR or CD87), which is associated with an aggressive cancer phenotype (Fig. 1G).

**Fig. 2:**
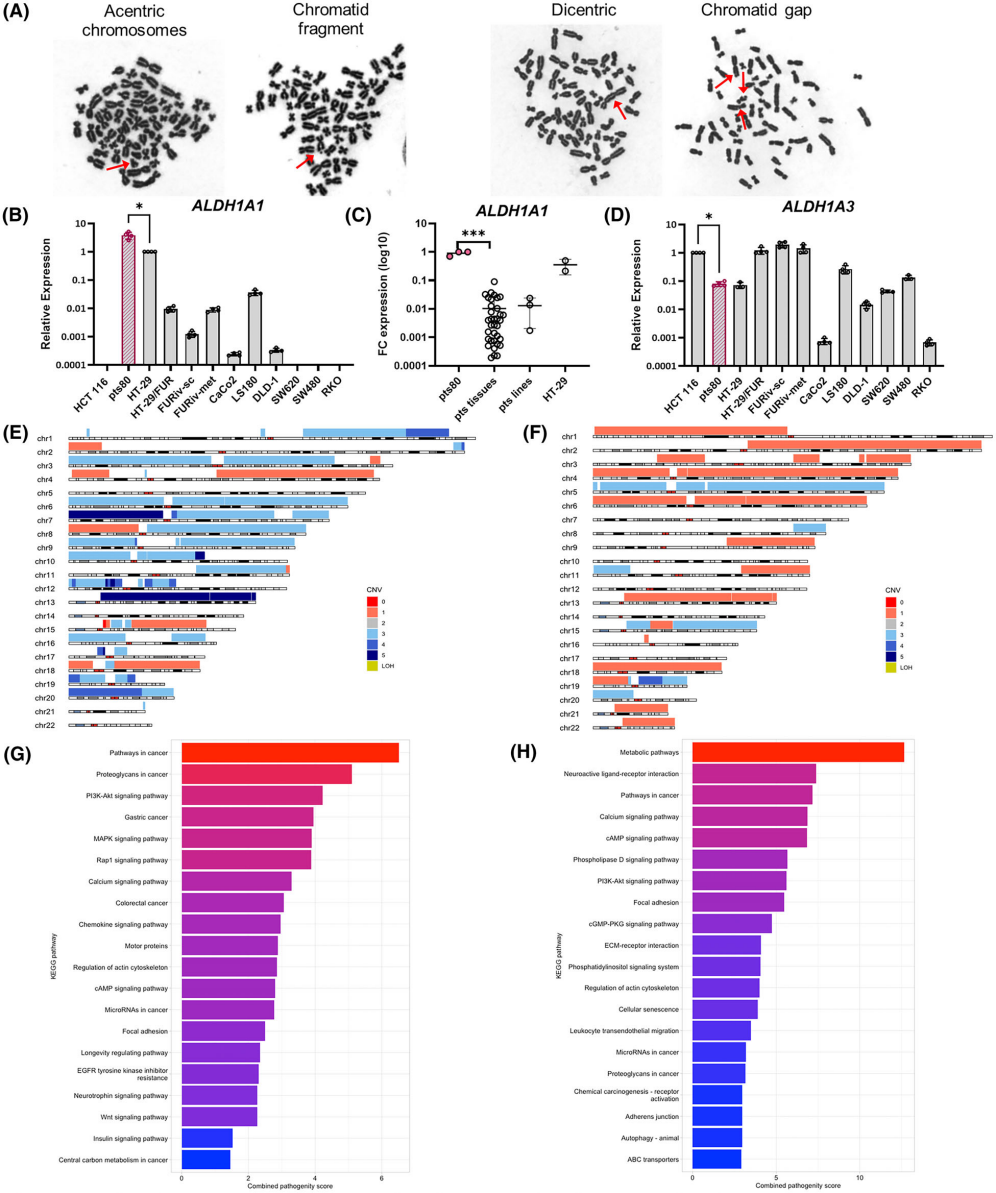
Characterization of the pts80 cell line: genomic and molecular properties. (A) Karyotype with chromosomal aberrations marked with arrows. Axio Imager Z2 fluorescence microscope (Carl Zeiss), Metafer 3.6 software (MetaSystems), magnification 630×, representative images of triplicates. (B, C) Fold change expression analysis of *ALDH1A1* mRNA among (B) CRC cell lines vs. HT‐29 cell line, (C) patient (pts) tumor tissues, and patient‐derived cell line with significant overexpression in pts80 cells. Each dot represents: pts tissues – 35 different patients' CRC tissues, pts lines – 3 different patient‐derived cell lines, 3 biological replicates of pts80 and HT‐29, analyzed by Mann–Whitney test. (D) Fold change expression analysis of *ALDH1A3* mRNA vs. HCT 116 line. (B, D) Gene expression was normalized to the *HPRT1* housekeeper, each dot represents the technical replicate (*n* = 4), RT‐qPCR, and results are representative of three independent experiments, error bars represent SD, analyzed by Mann–Whitney test. (E) Copy number variation (CNV) map of the patient's tumor tissue. (F) CNV map of the derived pts80 cell line; colors indicate the number of copies of DNA segments. (G) Significantly changed pathways with combined pathogenicity score based on somatic mutations of the patient's tumor tissue; WES, KEGG pathway database. (H) Significantly changed pathways based on somatic mutations of pts80 cell line; WES, KEGG pathway database (*P < 0.05, ***P < 0.001).

The cytogenetic analysis revealed aneuploidy with 58–62 chromosomes for every metaphase. In one hundred investigated metaphase cells, there were 61.43% of dicentric chromosomes, 7.85% of acentric chromosomes, 6.14% of chromosomal gaps, and 9.22% of chromosomal fragments (Fig. 2A). Overall, chromosomal aberrations were present in 84.64% of analyzed cells.

We compared the expression of ALDH1A1 and ALDH1A3 among a set of 12 CRC cell lines. The mRNA expression of the *ALDH1A1* gene was markedly the highest in the pts80 cell line, and the second highest was in the HT‐29 cell line. Although HT‐29 cells are known for significant upregulation of *ALDH1A1*, the established pts80 cell line exerted even 4.4 times higher *ALDH1A1* expression than HT‐29 (Fig. 2B). We compared *ALDH1A1* expression to 35 patients' tumor tissues of colon adenocarcinoma, rectal adenocarcinoma or CRC metastasis into the liver and to three other patient‐derived cell lines established in our laboratory. The tested patient‐derived cell lines have 42, 73, and 576 times lower *ALDH1A1* expression than pts80. Thirty‐five tumor tissues expressed ALDH1A1 at a significantly lower level than pts80 (FC is 11–5238 times lower than pts80, with a median of 252). This clearly indicates that pts80 is unique in ALDH1A1 overexpression (Fig. 2C). For analysis of *ALDH1A3* expression, the HCT 116 cell line was set as a reference cell line. The pts80 cell line exhibits 7.7 times lower *ALDH1A3* gene expression compared to the reference cell line (Fig. 2D).

Detailed genomic analysis was performed using CNV analysis and WES of the original tumor tissue (Fig. 2E) and the derived cell line (Fig. 2F). A classification of CNV into benign, uncertain significance, and pathogenic by Interpretation of Structural Variants (ISV) prediction was made. CNV analysis has shown numerous large‐scale genomic alterations in the original patient's tumor tissue, such as CNV gains spanning chromosomes 1, 3, 6, 7, 8, 9, 10, 11q, 13q, and 20 and CNV losses of chromosomes 4q, 8p, 15q, and chromosome 18 to single copy (Table 3, Fig. 2E). Overall, nearly 50% of the genome length was shown to be aberrant, while the vast majority of CNV findings were predicted pathogenic as they spanned numerous genomic elements, including genes associated with CRC malignant processes. From those, the gain of chr9:68500000–137 999 999 overlaps the *ALDH1A1* sequence in tumor tissue and thus increases the gene copy numbers.

**Table 3:** CNV of gDNA of original patient's tumor (Fig. 2E) and derived cell line (Fig. 2F). Chromosomes and parts of chromosomes not listed in the table are diploid.

No. of copies	CNV of the original patient's tumor	CNV of the derived pts80 cell line
5	Chr.7p, 13q	None
3–4	Chr.1 and 20	Ch.19q
3	Chr.3, 6, 7q, 8, 9, 10, 11q	Chr.5, 11p, 15q, 20p
1	Ch.4q, 8p, 15q, 18	Chr.1p, 2q, 4, 6, 9q, 13, 11q, 21, 22

WES analysis of the original tumor tissue, pts80 cell line, and peripheral blood mononuclear cells was performed. The blood cells were used to filter germline findings from somatic variants in the tumor and the pts80 cell line. Somatic variants were evaluated for their impact on coding and non‐coding genes and for their pathogenicity by combined pathogenicity score. Only variants that are predicted to cause high or moderate impact or variants evaluated by bioinformatics tools as likely pathogenic were selected. In the patient's rectal tumor, 27 variants (24 missense mutations and 3 stop codons) were selected as likely pathogenic, comprising 20 variants proven to be associated with CRC (Table 4). The somatic *TP53* mutation was detected (c.404G>A, p.C135Y) in tumor tissue, besides *BRAF* oncogene mutation (c.1397G>A, p.G466E) and *SMAD6* mutation (C.1427A>G, p.Y476C). Other CRC‐associated genes, such as *LZTS3* and *PDCD4* tumor suppressor genes, *CDH8, PCDH17*, and *NRXN2* adhesion molecules, are mutated in rectal tumor tissue (Table 2). Enriched pathways based on somatic mutations of the patient's tumor tissue showed that pathways characteristic for cancer, PI3K/Akt, MAPK, Rap1, Wnt, and others are dysregulated (Fig. 2G).

**Table 4:** Somatic mutations detected in the original tumor tissue analyzed as likely pathogenic. In the genes marked as “not known,” the link to CRC has not yet been proven, but they cannot be excluded because they are a binding partner to molecules linked to CRC or belong to the gene family linked to CRC carcinogenesis.

Gene	Gene function	HGVSc	HGVSp	Classif.	CRC association	Ref.
PDCD4	Tumor suppressor	c.886G>T	p.D296Y	Likely pathogenic	Prognostic marker for CRC	[69]
NRXN2	Cell adhesion molecule	c.3703G>A	p.G1235S	Likely pathogenic	Proliferation, invasion & migration of CRC cells	[70]
DHX37	RNA processing in cancer	c.1430C>T	p.T477M	Likely pathogenic	Highly enriched in critical cancer signaling pathways	[71]
GRIP1	Nuclear receptor coactivator 2, Wnt pathway	c.2782A>C	p.T928P	Likely pathogenic	TIF2/GRIP‐1/SRC‐2 downregulated in cancer tissues	[72]
ZDHHC17	Regulates the subcellular localization of proteins	c.1310T>G	p.I437R	Likely pathogenic	Not known, but ZDHHCs regulate CRC proliferation, invasion	[73]
XPO4	Protein export from the nucleus to the cytoplasm	c.697G>C	p.V233L	Likely pathogenic	Its mutation found in CRC tissues	[50]
ANKS1B	Overexpression in pre‐B cell ALL	c.2812C>A	p.R938S	Likely pathogenic	siRNA ANKS1B inhibited migration & invasion in CRC cells	[74]
SMAD6	Inhibition of Smad2/3/4 complex in TGFb pathway	c.1427A>G	p.Y476C	Likely pathogenic	Correlated with OS of CRC patients	[75]
NBEA	Neuronal ca	c.4861A>C	p.S1621R	Likely pathogenic	Its mutation found in CRC tissues	[76]
PCDH17	Cell adhesion molecule	c.2989A>C	p.T997P	Likely pathogenic	Increases the sensitivity of CRC to 5‐FU treatment	[77]
PCDH9	Cell adhesion molecule	c.978C>A	p.H326Q	Likely pathogenic	Not know, member of cadherins	
MDGA2	Regulation of presynapse assembly	c.2638G>A	p.E880K	Likely pathogenic	No	
**TP53**	Tumor suppressor	c.404G>A	p.C135Y	Likely pathogenic	Driver gene of CRC	
ZNF787	Transcription factor	c.271G>A	p.E91K	Likely pathogenic	Not known, but ZFP family is correlated with CRC	
LZTS3	Tumor suppressor	c.1079G>T	p.W360L	Likely pathogenic	Associated with poor CRC prognosis	[78]
CTNNBL1	Putative regulator of the Wnt	c.1564C>T	p.R522X	Likely pathogenic	SNPs in CTNNBL1 may be associated with CRC	[79]
KCNQ2	Potassium channel	c.911 T>A	p.F304Y	Likely pathogenic	No	
THSD7B	Angiogenesis	c.2359A>G, c.217G>A	p.R787G, p.G73R	Likely pathogenic	No	
**BRAF**	Oncogene	c.1397G>A	p.G466E	Likely pathogenic	Driver gene of CRC	
SLC34A2	Cotransporter involved in phosphate homeostasis	c.1622C>T	p.P541L	Likely pathogenic	Promotes cancer proliferation and cell cycle progression	[80]
URGCP	Activated NF‐κB pathway	c.796G>A	p.V266M	Likely pathogenic	Correlated with poor prognosis in CRC patients	[81]
UBE2D4	Protein ubiquitination	c.227C>T	p.P76L	Likely pathogenic	Not known, some ubiquitin‐proteasome enzymes are assoc.	
ADCY1	Catalyzes the formation of cAMP from ATP	c.3029A>T	p.D1010V	Likely pathogenic	Its mutations influencing drug effectiveness	[82]
LINGO2	Regulating axon growth and myelination	c.1503A>C	p.K501N	Likely pathogenic	TFF3/LINGO2 reverses tonic inhibition of EGFR	[83]
TCERG1L	Transcription factor	c.856+115C>T	Stop codon	Modified by mutation	Its hypermethylation is an early event in CRC	
**CDH8**	Adhesion, metastasis	c.1024‐40T>G	Stop codon	Modified by mutation	Driver gene of CRC	[84]
ST6GALNAC6	Sialyl transferase	c.27‐36A>G	Stop codon	Modified by mutation	Assoc. with CRC progression, migration, invasion	[85]

In line with the observed dysregulation of Wnt signaling, we performed a focused evaluation of genomic alterations affecting *APC*, as it is a key component of this pathway with a central role in colorectal tumorigenesis. Although WES did not identify pathogenic single‐nucleotide variants in *APC*, several alterations were detected in additional Wnt‐related genes, including a premature‐stop variant and an intronic change in *PPP3CB*, a synonymous variant in *WNT2*, and an upstream regulatory variant in *WNT3*. Importantly, copy number analysis revealed a large genomic gain spanning chr5:71.5–181.5 Mb that includes the *APC* locus. This region encompassed numerous coding and regulatory elements and fully overlapped the recurrent 5q35 Sotos syndrome region with established triplosensitivity (TS score = 3), which contains *NSD1*, a histone methyltransferase with context‐dependent transcriptional regulatory activity. These combined sequence‐level and structural alterations support the involvement of Wnt‐pathway deregulation in the pts80 genomic landscape.

The gDNA from the pts80 cell line carries 68 genes with somatic mutations detected as likely pathogenic or with high impact. From these mutated genes, 37 coding molecules have been proven to be linked to CRC (Table 5). According to the higher number of mutations in the cell line, we assume that the selected subpopulation of tumor cells grew out of the patient's tumor tissue and continued with the accumulation of additional mutations in the cell line.

**Table 5:** Somatic mutations detected in the pts80 cell line analyzed as likely pathogenic. In the genes marked as “not known,” the link to CRC has not yet been proven, but they cannot be excluded because they are a binding partner to molecules linked to CRC or belong to the gene family linked to CRC carcinogenesis.

Gene	Gene function	HGVSc	HGVSp	Classif.	Association with CRC	References
ABCB1	Efflux pump	c.3800A>G	p.Y1267C	Likely pathogenic	Multidrug resistance in CRC	[86]
AGTR1	G protein‐coupled receptor	c.394C>A	p.H132N	Likely pathogenic	Poor RFS in stage I‐III CRC patients	[87]
CDH7	Transcription factor	c.5390G>C	p.G1797A	Likely pathogenic	CRC tumor progression	[88]
COL6A3	Microfibrillar component of ECM	c.7820G>T	p.R2607M	Likely pathogenic	CRC poor prognosis	[89]
CPNE6	Ca‐dependent membrane‐binding protein	c.1610A>C	p.Q537P	Likely pathogenic	Not known, but CPNE7 promotes growth and EMT in CRC	[90]
CPPED1	Protein serine phosphate	c.564G>C	p.Q188H	Likely pathogenic	Neoantigen from stage IV CRC tissue	[91]
DYNC1I1	Part of the cytoplasmic dynein 1 complex	c.1015G>A	p.V339M	Likely pathogenic	Prognosis risk biomarker for CRC	[92]
EIF3F	Regulation of protein translation	c.287C>A	p.P96Q	Likely pathogenic	Upregulated in CRC tissues	[93]
EPHB3	Maintenance of intestinal crypt architecture	c.182G>C	p.G61A	Likely pathogenic	Overexpression assoc. with better clinical outcome of CRC	[94]
FCGR3A	Surface Fc gamma receptor 3a	c.538A>T	p.N180Y	Likely pathogenic	SNPs assoc.with clinical outcome of mCRC patients with anti‐EGFR mAb	[95]
FH	TCA cycle enzyme	c.1391G>T	p.G464V	Likely pathogenic	CRC poor prognosis	[96]
GRA4	Neurotransmitter receptor	c.1976A>C	p.E659A	Likely pathogenic	Its methylation is a CRC tissue‐specific biomarker	[97]
KIAA1549	Cell signaling, protein management	c.4807C>T	p.R1603W	Likely pathogenic	Promotes the proliferation and migration of CRC cells	[98]
KLK13 (PSA)	Serine protease	c.634G>T	p.D212Y	Likely pathogenic	Assoc. with OS in CRC patients	[99]
MIB1	Ubiquitination of Notch pathway's protein	c.1857A>T	p.L619F	Likely pathogenic	Often mutated in CRC tumors	[100]
MCOA4	Selective transport receptor	c.1322G>A	p.G441E	Likely pathogenic	Mediated ferritinopathy in CRC	[101]
NEURL1B	Methylation and microRNA target regulation	c.1534G>T	p.D512Y	Likely pathogenic	Its methylation is assoc. with poor survival in CRC	[102]
NOVA2	Regulation of alternative mRNA splicing	c.428G>C	p.G143A	Likely pathogenic	Expressed in CRC tumor vasculature	[103]
PCDH17	Tumor suppressor in CRC	c.3703G>A	p.G1235S	Likely pathogenic	Increases the sensitivity to 5‐FU treatment by inducing apoptosis	[77]
PECAM1	Adhesion molecule	c.550C>T	p.R184X	Likely pathogenic	β catenin‐mediated EMT in CRC	[104]
PKD1L2	Polycystin 1	c.2498 + 1G	Stop codon	High impact	Its CNVs are associated with CRC	[105]
RBFOX1	Alternative splicing of tissue‐specific exons	c.1106G>C	p.R369T	Likely pathogenic	Its mutations are in CRC cell lines and tumors	[106]
SLC2A13	Membrane transport protein	c.1231G>A	p.G411R	Likely pathogenic	Not known, but SLC12A2 is overexpressed in CRC tissues	
SLC30A10	Membrane transport protein	c.927G>T	p.M309I	Likely pathogenic	Not known	
SLC7A6	Membrane transport protein	c.1259G>C	p.R420P	Likely pathogenic	Not known	
SPAG9	JNK and MAPK pathways	c.3478G>T	p.V1160L	Likely pathogenic	Overexpressed in CRC tumors	[107]
SPATA13 (ASEF2)	Alternat. guanine nucleotide exchanging factor	c.2706C>A	p.F902L	Likely pathogenic	APC‐induced tumorigenesis	[108]
SPTBN2	Target of miR‐214‐3p	c.564G>T	p.M188I	Likely pathogenic	Overexpressed in some CRC cell lines	[109]
STRAP	Required for APC‐induced tumorigenesis	c.113A>T	p.D38V	Likely pathogenic	Wnt pathway activation	[110]
STX4	Regulation of synaptic vesicle exocytosis	c.131.2A>	Stop codon	Likely pathogenic	CRC metastasis, invasion & growth by increased exosomes secretion	[111]
STXBP5L	Binds STX4	c.711C>G	p.D237E	High impact	Not known, but binding partner STX4 is associated with CRC	[112]
TTN	Muscle protein	c.95264G>	p.W31755	Likely pathogenic	Overexpression assoc. with short OS in CRC	[113]
ZNF28	Transcription factor	c.1926G>T	p.K642N	Likely pathogenic	Not known, but ZNFs regulate CRC proliferation, EMT, stemness	[114]
ZNF33B	Transcription factor	c.1657T>A	p.F553I	Likely pathogenic	Not known, but ZNFs regulate CRC proliferation, EMT, stemness	
ZNF654	Transcription factor	c.2492A>C	p.Q831P	Likely pathogenic	Not known, but ZNFs regulate CRC proliferation, EMT, stemness	
ZNF749	Transcription factor	c.1768G>T	p.G590W	Likely pathogenic	Not known, but ZNFs regulate CRC proliferation, EMT, stemness	
ZNF836	Transcription factor	c.1887G>T	p.K629N	Likely pathogenic	Not known, but ZNFs regulate CRC proliferation, EMT, stemness	

The somatic mutations affected the transport proteins and receptors (*AGTR1, NCOA4*, genes coding Solute Carrier Family members 2A13, 30A10, 7A6), genes regulating protein transcription, translation, and post‐translational modification (*EIF3F, KIAA1549, NEURL1B, MIB1, RBFOX1*), multidrug resistance gene *ABCB1*, *Cadherin 7*, genes coding Zinc Finger Proteins, tumor suppressor *PCDH17*, adhesion molecule *PECAM1*. Some mutations affect genes involved in the Tricarboxylic acid cycle (*FH* as the gene for Fumarase) and Wnt, Jun, and MAPK signaling pathways (*SPAG9, SPATA13, STRAP*). Their name, function, mutation via HGVS nomenclature, and published association with CRC are in Table 5. Enriched pathways based on somatic mutations in the pts80 cell line are enlisted in Table S2 and Fig. 2H.

The chromosomal and mutational alterations mentioned above led to changes in the expression profile of the derived cell line compared to the original tumor tissue. The RNA sequencing revealed 3698 upregulated and 2676 downregulated differentially expressed genes (DEG; according to DESeq2 method; *P*‐value < 0.05, FC > 2). Gene set enrichment analysis and signaling pathways analysis revealed that p53/p21, IGF‐MAPK, PI3K/Akt, Hedgehog, JAK–STAT, Wnt, K‐Ras/Raf, Notch, VEGF, TGF‐β/Smad, EGFR and MSI signaling pathways are dysregulated in pts80 cell line. The complete list of DEG in the pts80 cell line vs. the patient's tumor tissue is in Table S3.

### 3.3 Downregulation of ALDH1A1 expression in the pts80 cell line using CRISPR‐Cas9

**Fig. 3:**
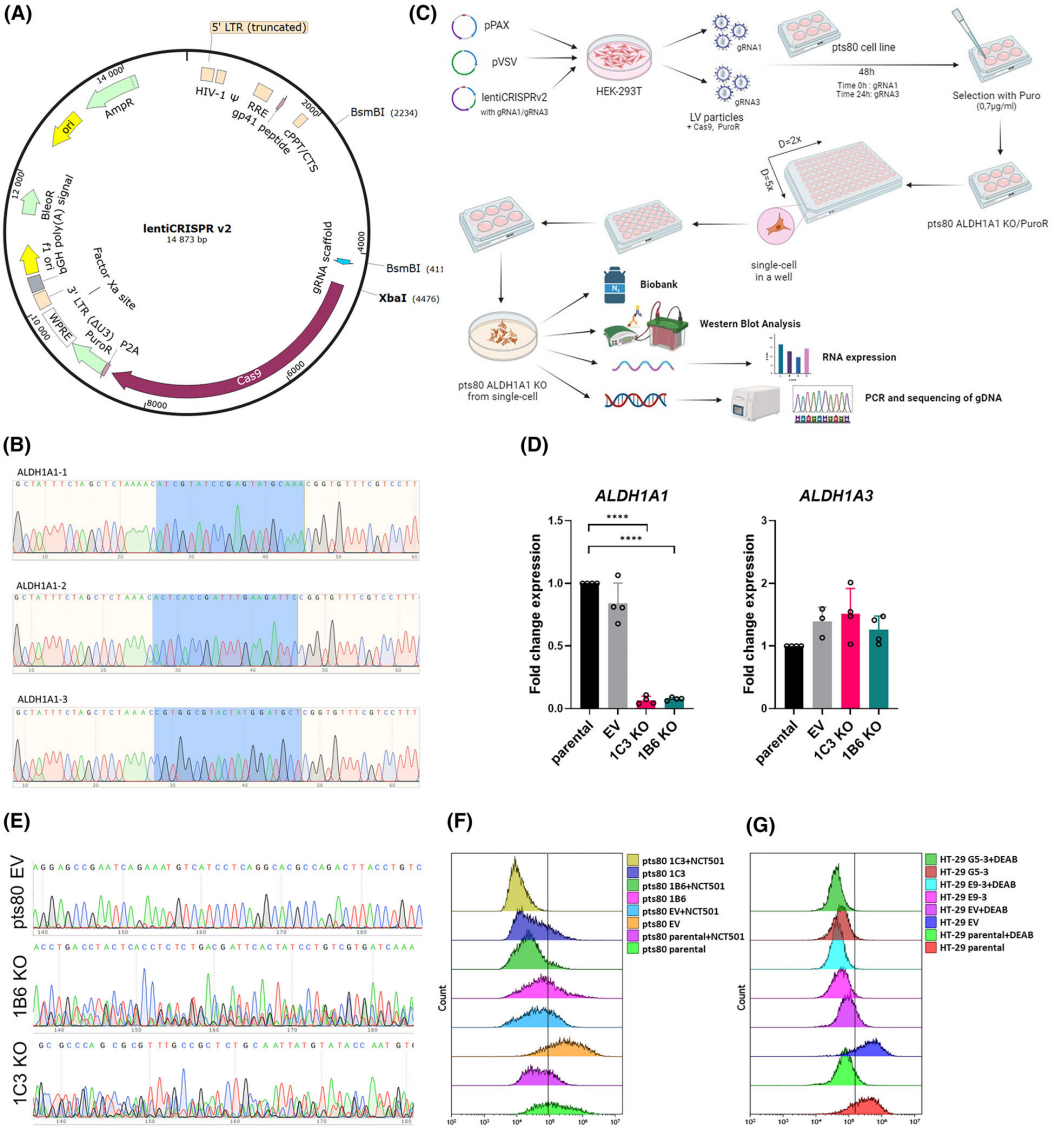
Design of CRISPR‐Cas9 *ALDH1A1* gene editing and characteristics of knockout clones 1B6 and 1C3. (A) lentiCRISPRv2 vector used for gene editing. (B) DNA sequences 1–3 contain specific guide RNAs for the *ALDH1A1* gene. (C) Method of preparation of knockouts, single‐cell‐derived clones, and their verification, created by Biorender. (D) Expression analysis of *ALDH1A1* and *ALDH1A3* mRNA in 1C3 and 1B6 clones vs. controls; RT‐qPCR, gene expression normalized to *HPRT1* housekeeper, analyzed by unpaired t‐test (*n* = 4), each dot represents the technical replicate, results from three independent experiments, error bars represent SD. (E) Chromatogram of *ALDH1A1* gene in knockouts contains overlapping sequences, confirmed in three independent experiments, Sanger sequencing. (F) Aldehyde dehydrogenase activity measured by ALDEFLUOR assay in pts80 cells; NCT‐501 ‐ specific ALDH1A1 inhibitor, results confirmed in three independent experiments. (G) Aldehyde dehydrogenase activity measured by ALDEFLUOR assay in HT‐29 cells; DEAB – ALDH inhibitor, results confirmed in three independent experiments (****P < 0.0001).

Two RNA sequences were chosen for the downregulation of ALDH1A1 expression in the pts80 cell line and cloned into lentiviral vector (Fig. 3A). Constructs were verified by sequencing (Fig. 3B). Our gRNAs targeted the promoter region and the start of the coding region. To increase the probability of achieving the knockout, a combination of lentiviruses generated by two different gRNAs (gRNA1 and gRNA3) was used for the transduction (Table 1). The pts80 cell line was also transduced with lentivirus generated by an empty vector, creating the transduction control (marked pts80 EV). All transduced cells were selected by puromycin before seeding single‐cell colonies (Fig. 3C).

Thirty different single‐cell‐derived colonies of ALDH1A1 knockouts were propagated, and the cell lysate of each colony was examined for the presence of ALDH1A1 protein by western blot. The parental pts80 cell line and pts80 EV control produced ALDH1A1 protein at a very high level. Two transduced and puromycin‐resistant clones (marked 1C3 KO and 1B6 KO) with significantly decreased ALDH1A1 production were chosen for further confirmation by *ALDH1A1* mRNA expression analysis (Fig. 3F) and Sanger sequencing (Fig. 3G). In comparison to the pts80 cell line (marked as the parental cell line in the following text), there was significantly decreased *ALDH1A1* expression in the 1C3 and 1B6 clones: for the 1C3 clone 6.38% and for the 1B6 clone 8.78% of expression in pts80 parental cells (Fig. 3F). This represents a 15.7‐fold reduction of ALDH1A1 expression in the 1C3 KO and 11.4‐fold in the 1B6 KO. The control pts80 EV cell line maintained high expression of *ALDH1A1* mRNA (88%) compared to the pts80 parental cell line, as demonstrated by RT‐qPCR.

For verification of genetic editing, the *ALDH1A1* gene was amplified using primers, including gRNA1 or gRNA3 sites, respectively. The amplified PCR products were visible as a double band after agarose gel electrophoresis, and the sequencing of the products revealed the sequential alteration in the *ALDH1A1* gene in 1C3 and 1B6 clones. gDNA of both clones was cut in the same place (188–197 nt, antisense strand, NM000689.4), where the gRNA1 sequence was bound (194–214 nt). We have identified the deletion of 9 nucleotides and the following sequence shift after deletion. The two or three different sequences overlap on chromatograms (Fig. 3G) because each allele can be targeted with different editing patterns as a result of non‐homologous end joining (NHEJ). In both clones, the original intact sequence is still present, so 1C3 and 1B6 clones are incomplete knockouts (KO) with attenuated ALDH1A1 expression.

Besides, the HT‐29 cell line was transduced with the same lentiviruses carrying gRNA1 and gRNA3 targeting *ALDH1A1*. All transduced cells were selected by Puromycin and propagated from single‐cell colonies the same way as pts80 (Fig. 3C). Sanger sequencing of HT‐29 after CRISPR‐Cas9 editing confirmed the deletion of 34 base pairs of the *ALDH1A1* gene in both clones HT‐29 G5‐3 KO and HT‐29 E9‐3 KO. The alterations in gDNA were followed by a thousand‐fold decline of mRNA expression verified by RT‐qPCR: HT‐29 G5‐3 KO displayed 0.06% and E9‐3 KO 0.019% of the *ALDH1A1* expression of parental cells.

Subsequently, ALDH activity was determined to confirm the attenuation of enzymatic function. Due to significant overexpression of ALDH1A1 in pts80 cell line and multiplying the chromosome encoding the *ALDH1A1* gene, we modified the protocol by using the specific ALDH1A1 inhibitor NCT‐501 instead of DEAB in the control sample. We confirmed that knockout clones have a significantly reduced ALDH activity (Fig. 3D). The ALDEFLUOR™ assay confirmed the significantly reduced ALDH activity also in HT‐29 KO clones (Fig. 3E).

Additionally, we have examined the expression of other ALDH isoforms in pts80 and HT‐29 lines by RT‐qPCR to explore the possibility of genetic compensation of the downregulated ALDH1A1 isoform by other ALDH isoforms (Table 6). In pts80, there is no other ALDH isoform significantly overexpressed in both clones with attenuated ALDH1A1 (FC > 2). In HT‐29 knockouts, ALDH3A1 is upregulated compared to parental cells.

**Table 6:** Expression of ALDH isoforms in ALDH1A1 knockouts compared to its parental counterpart (RT‐qPCR) (ND ‐ not detected).

ALDH1 isoform	Fold change expression in pts80				Fold change expression in HT‐29			
	Parental	EV	1C3 KO	1B6 KO	Parental	EV	G5‐3 KO	E9‐3 KO
*ALDH1A1*	11	0.92	0.12	0.11	1	1.77	0.001	0.0001
*ALDH1A3*	11	1.30	1.10	1.12	1	0.87	0.29	0.08
*ALDH2*	11	0.67	1.33	1.19	1	0.68	0.23	0.23
*ALDH3A1*	11	1.32	1.01	0.77	1	0.76	2.65	2.31
*ALDH1B1*	11	0.71	0.46	0.84	1	0.87	0.50	0.69
*ALDH1A2*	11	3.01	1.29	0.35	ND	ND	ND	ND

### 3.4 Attenuation of ALDH1A1 enhanced the invasiveness and suppressed the clonogenic potential *in vitro*

**Fig. 4:**
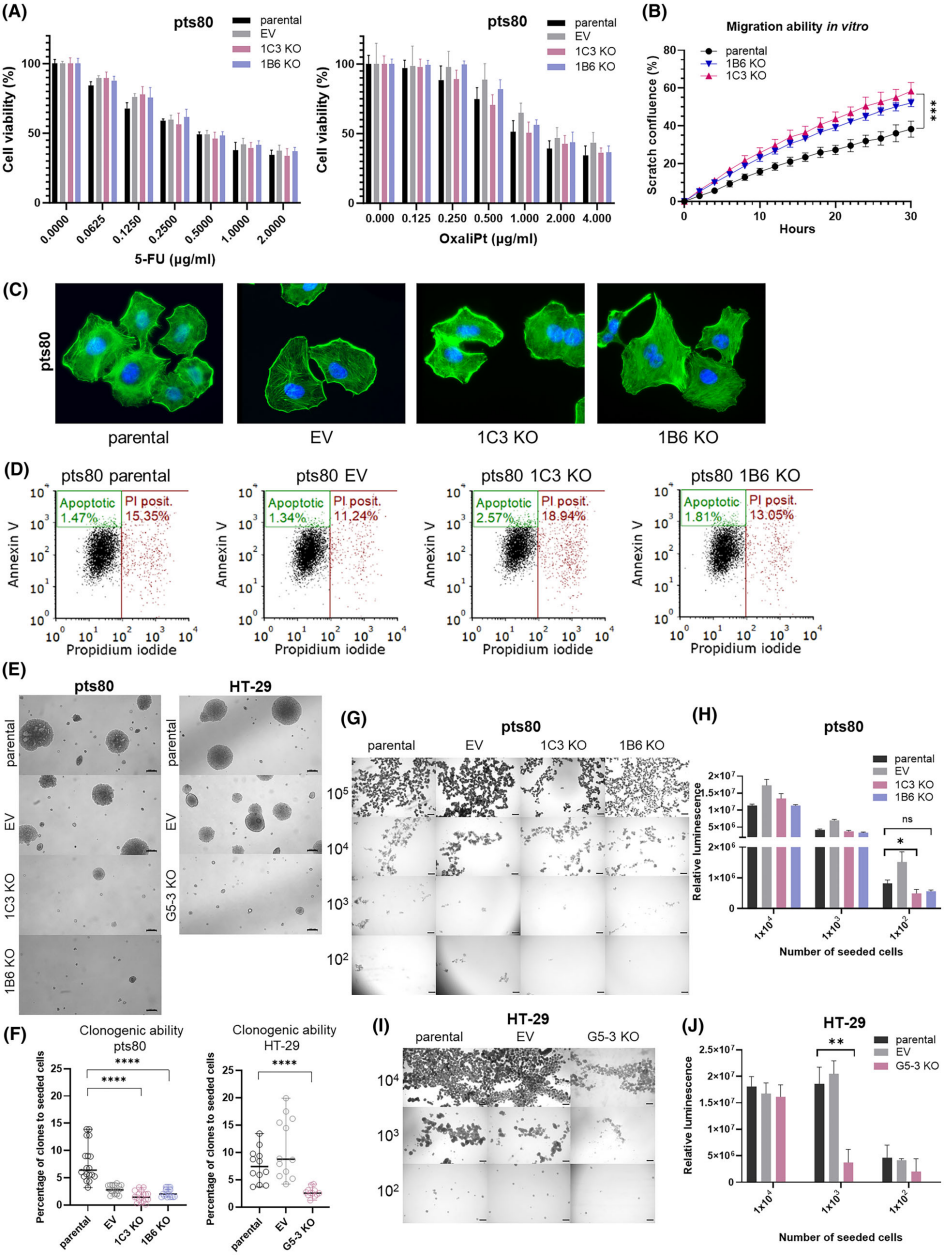
Biological properties of *ALDH1A1* knockouts *in vitro*. (A) Response to 5‐fluorouracil (5‐FU) and oxaliplatin (OxaliPt), analyzed by unpaired *T*‐test (*n* = 6), representative results of three independent experiments, mean ± SD. (B) Migration ability, scratch wound assay (*n* = 10), representative results of three independent experiments, mean ± SD, analyzed with paired Wilcoxon test. (C) Immunofluorescent staining of Actin filaments (Alexa Fluor 488 Phalloidin) and nuclei (DAPI). Axio Imager Z2 fluorescence microscope (Carl Zeiss), magnification 630×, Metafer 3.6 software (MetaSystems). (D) Detection of apoptosis, Annexin V staining, flow cytometry (*n* = 3). (E, F) Colony‐forming assay in soft agar. (E) Representative pictures of pts80 and HT‐29 colonies and their size, magnification 50×, ZEISS Axiovert microscope 1.0, scale bar: 100 μm. (F) Capability of pts80 and HT‐29 cells and their engineered counterparts to proliferate and form one cell‐derived colonies in soft agar, calculated as a ratio of growth colonies and seeded cells × 100% (*n* = 12 for HT‐29, *n* = 18 for pts80), each dot represents individual value, analyzed by Mann–Whitney test. (G–J) Colonosphere‐forming assay. (G, I) Representative images of single cell‐derived colonies, prepared from pts80 cells and HT‐29 cells, magnification 25×, Leica microscope Mateo/FL, scale bar: 400 μm. (H, J) Relative luminescence corresponding to viable cells proliferating in non‐adherent conditions as colonospheres. Assay was performed in triplicates, t‐test was used for statistical analysis, mean ± SD (*P < 0.05, **P < 0.01, ***P < 0.001, ****P < 0.0001, ns—not significant *P* ≥ 0.05).

The biological properties of pts80 1C3 and 1B6 clones with attenuated ALDH1A1 expression were compared to their control counterparts with high ALDH1A1 expression– parental cell line and pts80 EV control. As mentioned above, the parental cell line was sensitive to 5‐FU and oxaliplatin (IC_50_ = 0.49–0.54 and 1.08–1.54 μg·mL^−1^, respectively). Attenuation of ALDH1A1 in 1C3 KO and 1B6 KO clones did not change it; both clones were sensitive to 5‐FU (1C3: IC_50_ = 0.37–0.58 μg·mL^−1^, 1B6: IC_50_ = 0.51–0.65 μg·mL^−1^) and oxaliplatin (1C3: IC_50_ = 0.88–1.58 μg·mL^−1^, 1B6: IC_50_ = 1.28–1.88 μg·mL^−1^) (Fig. 4A).

The migration ability of pts80 1C3 and 1B6 KO tested by scratch wound healing assay showed that both knockouts had increased ability to migrate in comparison to the parental cell line (Fig. 4B). We did not perform this assay with the HT‐29 cell line because adherent culture does not proliferate in the compact monolayer that is essential for the migration assay. The ALDH1A1 attenuation did not influence the viability, morphology, or cytoskeletal structure of cells (Fig. 4C) and did not induce apoptosis measured by Annexin V staining (Fig. 4D). To better characterize the stem cell properties of ALDH1A1 knockouts, two *in vitro* assays were performed: a soft agar colony formation assay and a colonosphere‐forming assay. The ability of transformed cancer cells to grow and form colonies in an anchorage‐independent manner is a hallmark of CSCs. All cells formed colonies in soft agar, but the 1C3 KO and 1B6 KO colonies were apparently smaller compared to the large colonies from pts80 controls. Similarly, parental HT‐29 and HT‐29 EV control cells formed colonies, but the ALDH1A1 KO clone formed fewer and smaller colonies (Fig. 4E).

Only colonies larger than 30 cells were counted, and clonogenic ability was measured as the ratio of colonies to seeded cells. For the pts80 line, 6.4% of cells formed colonies from a single cell compared to 1.4% of 1C3 KO and 2.0% of 1B6 KO. Regarding the HT‐29 cell line, 7.4% of cells have colony‐forming potential compared with only 2.6% of G5‐3 KO (Fig. 4F). Culturing in serum‐free, non‐adherent conditions followed findings with cultures in soft agar. Both cell lines and their engineered counterparts formed single‐cell‐derived spheroids. Parental and EV pts80 cells proliferated in loose, irregular colonies. 1C3 KO‐derived colonospheres looked more compact and round‐shaped (Fig. 4G). A luminescence assay also revealed decreased proliferation of 1C3 KO cells compared to parental and EV cells (Fig. 4H). Parental, HT‐29 EV, and ALDH1A1 KO colonospheres were round‐shaped and compact (Fig. 4I). Decreased proliferation of the G5‐3 KO clone was observed and confirmed by decreased luminescence signal corresponding to lower number of proliferating cells (Fig. 4J). In both pts80 and HT‐29 cell lines, *ALDH1A1* attenuation affected and decreased the clonogenic ability of knockouts *in vitro*.

### 3.5 ALDH1A1 gene attenuation in the pts80 cell line is associated with enhanced metastatic potential *in vivo*

**Fig. 5:**
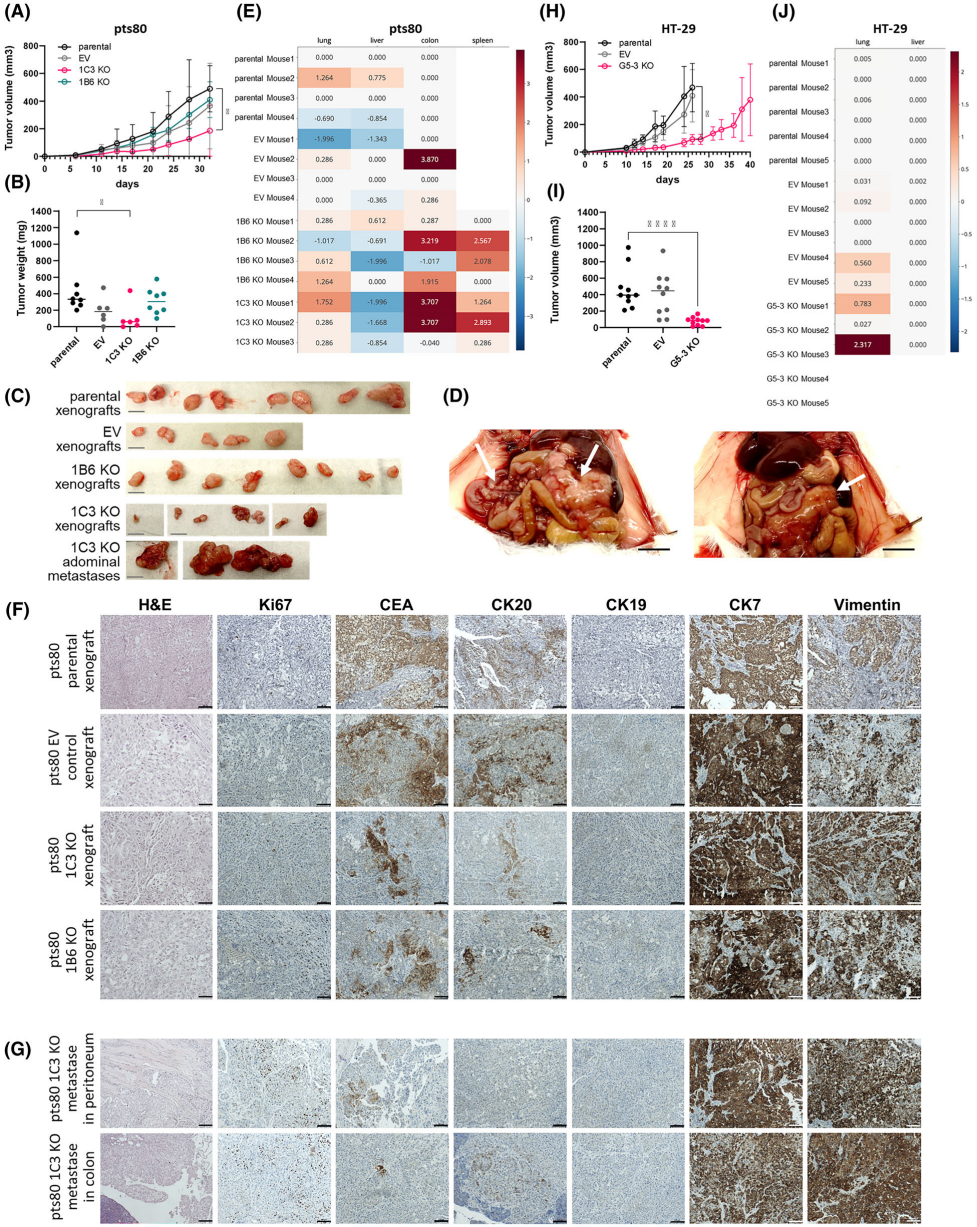
Tumorigenic and metastatic potential of *ALDH1A1* knockouts. (A) Growth of pts80 subcutaneous xenografts in time (2 × 10^6^cells, *n* = 8 for pts80 parental and 1B6 KO group, *n* = 6 for pts80 EV and 1C3 KO group due to the death of one mouse before the experiment ended). Tumor growth over time was analyzed by the Wilcoxon test, mean ± SD. (B) Weight of excised pts80 xenografts at the endpoint, values represent means ± SD (*n* = 6–8 as described above), analyzed by Mann–Whitney test. (C) Subcutaneous pts80 tumors and abdominal metastases at endpoint, scale bar: 1 cm. (D) Autopsy of the mouse with large colon and peritoneal pts80 1C3 KO‐derived metastases marked with arrows, scale bar: 1 cm. (E, J) qPCR quantification of pts80 (E) and HT‐29 (J) metastases in mouse organs from each mouse in each group, heat map displays nanograms of human DNA (logarithmic scale) in mouse tissue (absolute quantification by AriaMx Real‐Time PCR System), values represent means of technical replicates ± SD (*n* = 3). (F) Hematoxylin–eosin staining (H&E) and immunohistochemistry of pts80 s.c. xenografts for Ki67, carcinoembryonic antigen (CEA), cytokeratins 20, 19, 7 (CK20, CK19, CK7), and Vimentin. (G) H&E and immunohistochemistry of abdominal metastases of pts80 1C3 KO cells. (F, G) magnification 100×, Olympus microscope BX46, scale bar: 100 μm. (H) Growth of HT‐29 subcutaneous xenografts in time (1 × 10^5^ cells, *n* = 10 for each group). Mice with HT‐29 xenografts were euthanized on day 26. Tumor growth in time was analyzed by the Wilcoxon test, mean ± SD. (I) Tumor volume of HT‐29 xenografts on day 26 was analyzed by the Mann–Whitney test, values represent means ± SD (*n* = 10) (*P < 0.05, ****P < 0.0001).

Tumorigenic and metastatic potential of pts80 parental and engineered cells were tested on SCID/bg mice. One mouse from the EV group and one mouse from the 1C3 group died due to unspecified reasons during the experiment. Both parental and engineered cells formed subcutaneous xenografts. Growth dynamics of 1C3 KO tumors were significantly lower (*P* = 0.0078; paired two‐tailed Wilcoxon test) (Fig. 5A). On day 34, the parental and EV xenografts reached the endpoint size, and all experimental groups were sacrificed. The 1C3 KO xenografts displayed significantly lower volume and weight in comparison to parental xenografts. Tumors induced by 1B6 KO cells were smaller than xenografts from pts80 parental and pts80 EV controls, but the difference was not significant (Fig. 5B,C).

All mice underwent autopsy examination. In 4/4 mice with parental and 2/3 mice with EV controls, there were no signs of metastatic dissemination. The lungs and liver of every mouse were collected for routine checkout of micrometastases. During the autopsy, in one mouse of the pts80 EV group, suspicious changes in the colon were observed, and the sample was collected. In the 1C3 KO group, multiple abdominal macrometastases and micrometastases were found to cover the colon and peritoneum in all mice. Two 1C3 KO mice had 1330 mg and 2879 mg tumor mass in their abdomen (Fig. 5C,D). In all four 1B6 mice, the suspicious masses covering the peritoneum and colon were detected, but there was no large tumor mass, as in the 1C3 mice. In 1C3 and 1B6 mice, samples of colon, spleen, and abdominal mass, together with the lung and liver of every mouse, were collected. The presence of disseminated human cells in mouse organs was evaluated by quantitative PCR. The standard curve from gDNA extracted from the pts80 patient's peripheral blood cells was used for absolute qPCR quantification of human‐specific β‐globin gene copies in mouse tissues. The human β‐globin was confirmed in almost all analyzed tissues in groups 1C3 KO and 1B6 KO: lung, liver, spleen, colon, and tumor masses in the peritoneum (Fig. 5E). It was evident that 1B6 KO and 1C3 KO cells gained strong metastatic potential. In the pts80 parental control group, two mice were weakly positive for the human β‐globin in the lungs and liver, and two mice were free from metastases, showing 50% metastasis penetrance; in the pts80 EV group, two mice were free from metastases, two were weakly positive for the β‐globin gene in the spleen and lung, and one of them had a metastasis in the colon. Penetrance of metastasis for the pts80 EV group was similar to the pts80 parental group (50%) and differed from the 1C3 and 1B6 KO groups (100%).

Xenografts (Fig. 5F) and mouse tissues (Fig. 5G) were subjected to histological and immunohistochemical examination. Histological examination did not show significant differences among the xenografts from the parental pts80 cell line and ALDH1A1 knockouts. However, the differences between the original tumor and pts80 parental representative xenografts were detected (Table 7). The original tumor consisted mainly of oval cells with high uniformity in shape (80%), with primarily glandular arrangement, and it contained 70% of tumor cells and 30% of interstitial tissue. In comparison, pts80 cell line‐derived xenografts were composed of four different shapes (oval, spindle, polygonal, atypical) with minimum interstitial tissue. Moreover, the tubular and reticular arrangements together dominate over the glandular in the original tumor's cells, suggesting the drift to a low differentiated carcinoma in pts80 cell line‐derived xenografts.

**Table 7:** The histological comparison of the original tumor vs. xenografts from the pts80 parental cell line. Tumor cell arrangement, the ratio of necrosis, the ratio of tumor cell vs. interstitial tissue in each sample, and the description of tumor cell uniformity were compared. Types of cell shapes are marked as 1—oval/ovoid, 2—spindle, 3—polygonal, and 4—atypical, and the percentage means the ratio of cells with such shapes.

Sample	Tumor cell arrangement			Necrosis	Sample area		Tumor cell uniformity	
	Glandular	Tubular	Reticular		Tumor	Interstitial tissue	Size	Shape
Original tumor	70%	30%	0%	5%	70%	30%	Slight differences	1 (80%), 2 (20%), 3 (0%), 4 (0%)
Xenograft 1	50%	20%	30%	10%	90%	10%	Slight differences	1 (30%), 2 (40%), 3 (20%), 4 (10%)
Xenograft 2	50%	20%	30%	10%	90%	10%	Slight differences	1 (30%), 2 (30%), 3 (20%), 4 (20%)

The pts80 parental xenografts were described as non‐differentiated colorectal adenocarcinoma, positive for CEA and CK20. In contrast to the primary patient's tumor tissue histopathology (Fig. 1A), xenografts derived from the pts80 cell line displayed significantly decreased proliferation activity (Ki67 positivity) to 10%, which can be associated with cell dormancy. The positivity for cytokeratins CK20 and CK19 decreased (CK20 to 20–40%, CK19 close to 0%) in pts80 parental cell xenografts in comparison to the primary patient's tumor. The pts80 xenograft gained 100% positivity to CK7 and 50% to Vimentin. The pts80 EV subcutaneous xenografts and ALDH1A1 knockout 1C3 KO and 1B6 KO xenografts displayed similar immunohistochemical characteristics as the pts80 parental tissues: Ki67 marker of cell proliferation positivity dropped down to 5%, Vimentin positivity 100%, CEA 20%, CK20 20%, CK19 0%, and positivity CK7 100% (Fig. 5F). The 1B6 and 1C3‐derived metastases showed similar immunohistochemical characteristics as their corresponding subcutaneous xenografts (Fig. 5G). The increase of Vimentin positivity in 1C3 KO and 1B6 KO xenografts and metastases could indicate the EMT.

Based on the performed *in vitro* and *in vivo* analysis, we can conclude that the attenuation of the *ALDH1A1* gene in 1C3 and 1B6 clones shifted the colorectal cancer cells to a more invasive and metastatic phenotype.

These findings were confirmed by *in vivo* experiments performed on HT‐29 G5‐3 KO, compared to HT‐29 controls. The tumorigenicity of HT‐29 G5‐3 KO declined rapidly (xenografts reached 1cm^3^ after 40 days) in comparison to HT‐29 parental cell and HT‐29 EV ctrl (xenografts reached 1cm^3^ after 26 days) (Fig. 5H,I). Analysis of human B‐globin sequences revealed the presence of micrometastasis in 5 lungs out of 5 injected mice with HT‐29 G5‐3 KO compared to 2 positive lungs in HT‐29 cell line and 2 positive lungs in HT‐29 EV ctrl out of 5 injected mice for each group. One liver in HT‐29 EV ctrl was slightly positive (on the edge of the detection limit); livers of all other mice were negative for micrometastasis (Fig. 5J). The difference in micrometastasis penetrance in HT‐29 supports the findings of aggressiveness connected to ALDH1A1 attenuation.

The frequency of tumor‐initiating cells (TICs) or CSCs within a cell population is mostly quantified by the limiting dilution tumor initiation assay, which evaluates the self‐renewal capacity of cancer cells *in vivo*. Genetic editing of ALDH1A1, as a known stem cell marker, could modify the TICs frequency of the pts80 and HT‐29 line.

**Fig. 6:**
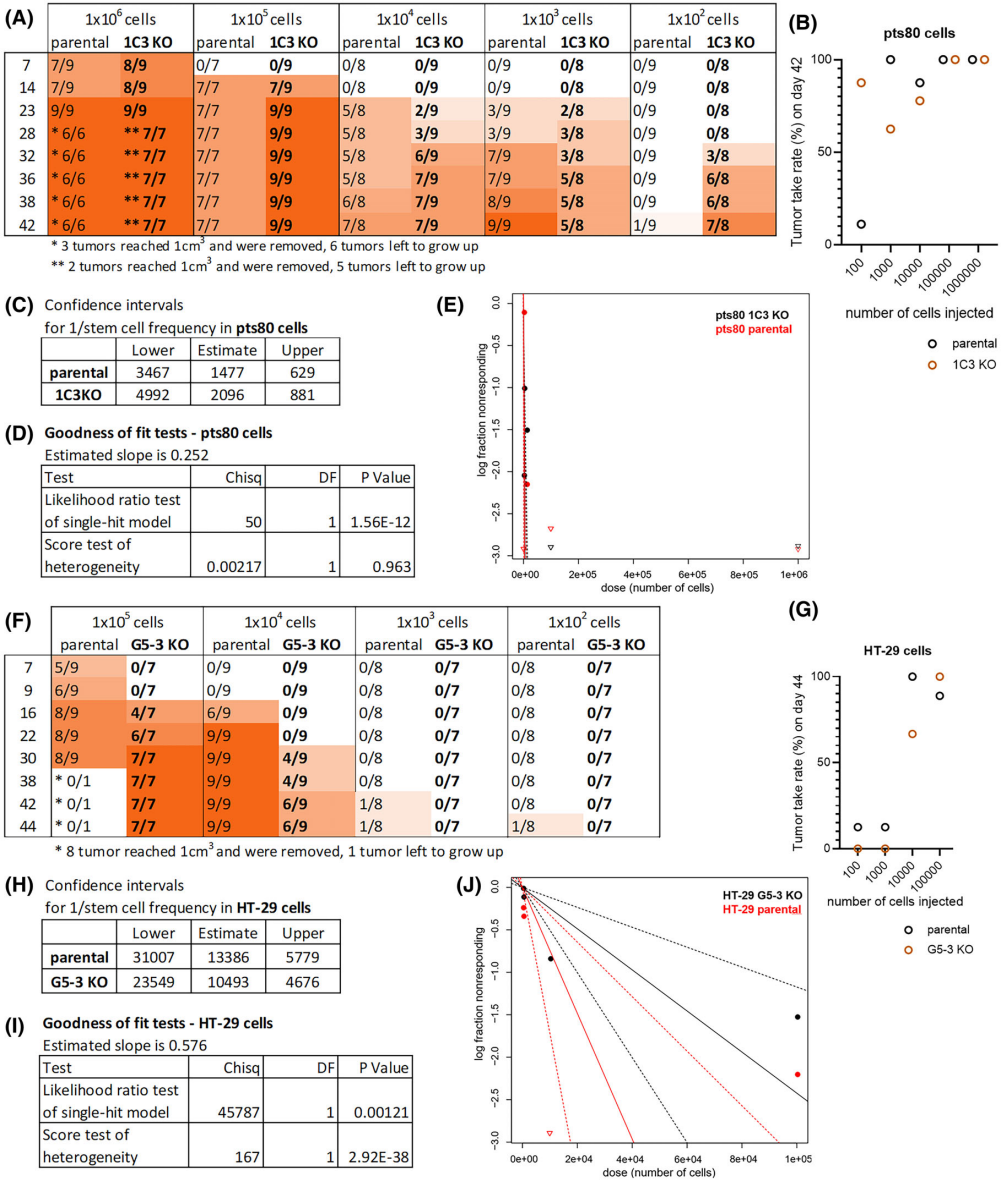
Analysis of the frequency of tumor‐initiating cells of ALDH1A1 knockouts by Extreme limiting dilution assay *in vivo*. Data were obtained from a single *in vivo* experiment; nine subcutaneous tumor cell inoculations per group were performed. (A, F) Tumor take rates of pts80 parental line and 1C3 KO (A) and HT‐29 line and G5‐3 KO (F) at different time points after subcutaneous application. (B, G) The plot shows the ratio of palpable tumor to the number of s.c. applications at the endpoint for each cell number. (C, H) Estimation of stem cell frequency and confidence intervals for pts80 and 1C3 KO (C) and HT‐29 cells and G5‐3 KO (H). (D, I) Statistics of single‐hit test and test of heterogeneity for pts80 (D) and HT‐29 (I). A higher Chi‐square score and lower *P*‐value (<0.05) indicate that the tumor did not grow from one cell. Single‐hit model is highly unlikely in pts80 cells. Test of heterogeneity: a higher χ^2^ and a lower *P*‐value mean the heterogeneity and variability between groups, for example, in HT‐29 cells. (E, J) The plot shows the direct relationship between the number of injected cells (*X* axis) and the logarithm of the fraction of mice without a tumor (*Y* axis). (E) For pts80, there is no direct proportionality between cell number and tumor take rates. (J) For HT‐29, there is a direct relationship between cell number and tumor take rate.

To compare the frequency of TICs in parental pts80 cell line and its ALDH1A1‐edited counterparts, a gradually decreasing number of pts80 parental and 1C3 KO cells (from 10^6^ to 10^2^ cells) were injected subcutaneously. At higher cell numbers (10^6^, 10^5^), palpable tumors appeared almost simultaneously; however, 1C3 KO were slightly smaller in volume than the pts80 parental. The xenografts of 1C3 KO (10^4^, 10^3^ applied cells) were palpable with a delay of several days after the pts80 parental. However, 87.5% xenografts grew sooner from one hundred applied 1C3 KO cells compared to one xenograft (11.1%) from the pts80 parental till the end of the experiment. The xenograft take rates at specific time points are shown in Fig. 6A. The tumor take rates at Day 42 (endpoint) for both groups (Fig. 6B) were used to calculate TICs frequency. The frequency of the TICs was determined to be 1 to 13 386 for the pts80 parental cell line and 1 to 10 493 for 1C3 KO (Fig. 6C) (χ^2^ = 0.691, DF = 1, *P* = 0.406), meaning no significant difference in the frequency of TICs. The results suggest that either more than one cell is required to produce a xenograft (estimated slope = 0.252, Single‐hit model negative) (Fig. 6D). There is no direct relationship between the number of cells and the tumor take rates (Fig. 6E).

Based on previous results, we knew that 10^5^ HT‐29 cells had formed a xenograft with 100% penetrance; 10^5^ to 10^2^ HT‐29 and G5‐3 KO cells were applied. HT‐29 xenografts formed by 10^5^ and 10^4^ cells applied, the tumor burden was significantly higher in xenografts from the HT‐29 parental cell line. HT‐29 G5‐3 KO xenograft appeared 10–14 days after the detection of HT‐29 parental line's xenografts (Fig. 6F). G5‐3 KO also formed smaller xenografts in volume and weight compared to the same amount of HT‐29 parental cells. For the 10^3^ and 10^2^ cell dosages, no xenografts were detected in HT‐29 G5‐3 KO group, while one tumor for both dosages was initiated by HT‐29 cells (Fig. 6F). The tumor take rates at Day 44 (endpoint) (Fig. 6G) were used to calculate TICs frequency. The frequency of the tumor‐initiating cells was determined to be 1–13 386 for the HT‐29 cell line and 1–10 493 for the HT‐29 G5‐3 KO (Fig. 6H) (χ^2^ = 0.0215, DF = 1, *P* = 0.643), so there is no significant change in the frequency of tumor‐initiating cells. Formation of the tumors could be a result of more than a single cell (estimated slope 0.576, Single‐hit model, Chisq = 10.5, *P* = 0.00121, Fig. 6I). For HT‐29 cells, a higher number of cells gives a higher frequency of tumors (Fig. 6J). The genetic attenuation of ALDH1A1 did not directly alter the tumor‐initiating ability *in vivo* of either pts80 or HT‐29, even though there were differences in xenograft growth specific to each cell line.

### 3.6 Changes in CNV profile between pts80 ALDH1A1 knockouts and pts80 parental cell line

**Fig. 7:**
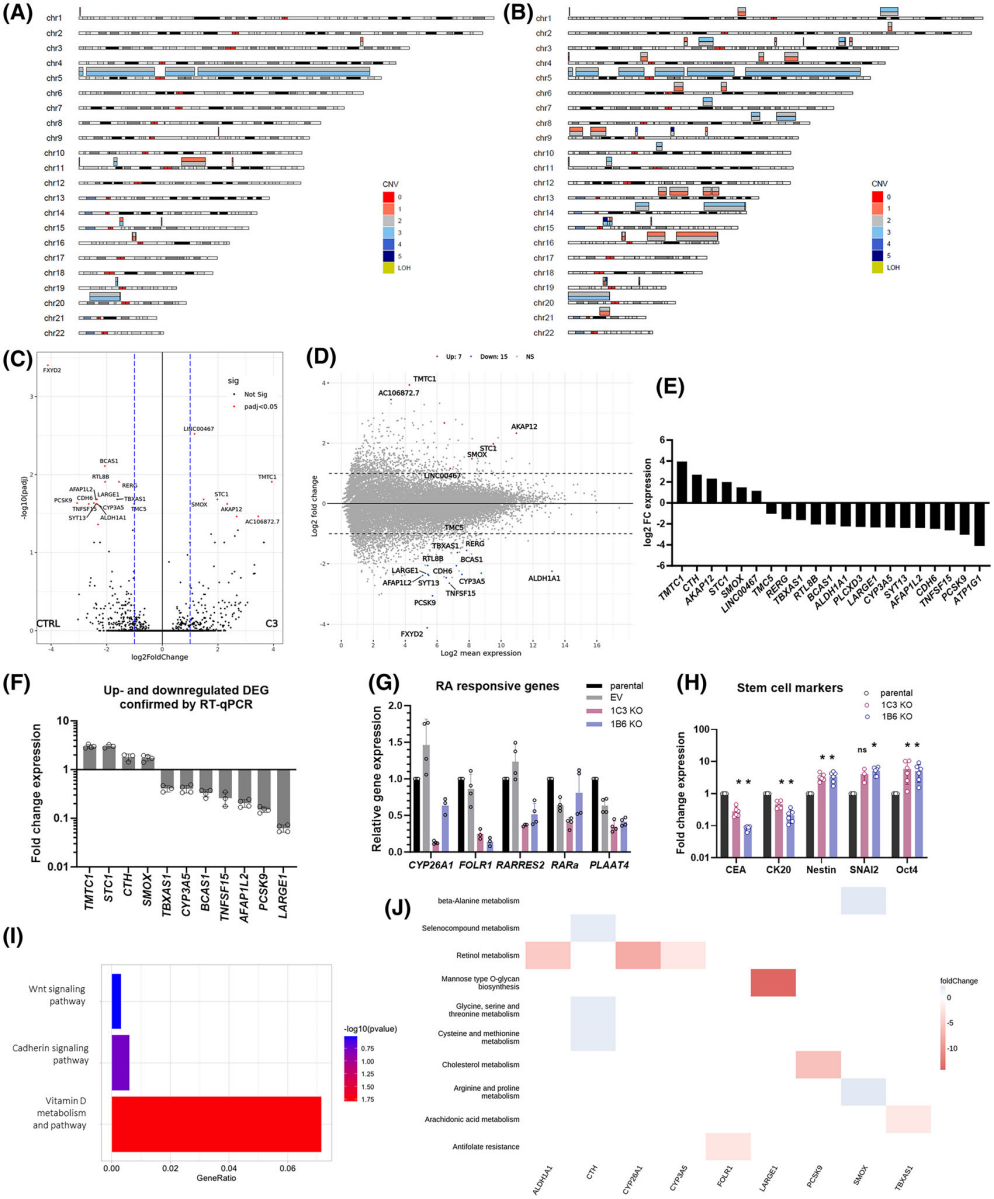
Genomic and molecular characteristics of pts80 ALDH1A1 knockouts. (A, B) Comparison of CNV profile between 1B6 KO (A) and 1C3 KO (B) vs. pts80 parental cell line. The aberrations are indicated as colored rectangles above the reference genome; the upper represents 1B6 KO (A)/1C3 KO (B), and the lower represents the pts80 parental cell line. Copy numbers (ranging from 0 to 5 copies) of the genomic region are differentiated by the color scale. (C) DEG between 1C3 KO vs. parental pts80 cell line, Volcano plot, RNAseq, genes with the most significant changes in expression (based on DESeq2 *P*‐values; *Y* axis) are listed. (D) DEG in 1C3 KO vs. pts80 parental cell line, MA plot, RNAseq, genes with the highest expression changes are at higher levels (*X* axis). (E) The plot of fold change (FC) expression of DEGs between 1C3 KO and pts80 parental cells, RNAseq. (F) Selected cancer‐associated DEG confirmed by RT‐qPCR in 1C3 KO vs. parental cell line, error bars represent SD. (G, H) RT‐qPCR expression of retinoic acid (RA)‐responsive genes (G) and stem cell markers (H) in KOs vs. parental cell line; (F–H) fold change in gene expression normalized to *HPRT1* housekeeper, analyzed by unpaired *T*‐test (F, G) or Wilcoxon test (H), each dot represents the technical replicate (*n* = 4), error bars represent SD, RT‐qPCR was performed in three independent experiments. (I) Gene enrichment analysis from previously confirmed DEGs between 1C3 KO vs. parental cells. (J) Enriched metabolic pathways associated with DEGs between 1C3 vs. parental cells (*P < 0.05, ns – not significant *P* ≥ 0.05).

CNV analysis of pts80 1B6 (Fig. 7A) and 1C3 clones (Fig. 7B) has shown several similarities with parental cells, suggesting that ALDH1A1 knockouts maintain aberrant profiles to a large extent. However, several differences in the copy numbers of specific DNA segments have been observed. Compared to pts80 parental cells, the 1B6 knockouts have shown a decrease in the numbers and length of detected CNVs, whereas in 1C3 knockouts, the number of aberrations increased (Fig. 7B). In both 1C3 and 1B6 cells, numerous variants span the same region as the aberrations in parental cells but primarily affect shorter stretches of DNA. The ALDH1A1 attenuation in knockout cells leads to the normalization of the copy numbers in some previously aberrant genomic regions.

### 3.7 ALDH1A1 attenuation leads to gene expression changes, promoting invasiveness and reducing cell differentiation

Total RNA from pts80 parental cell line, EV control, 1C3 KO, and 1B6 KO was used for the RNA library preparation and subjected to RNA sequencing. RNA was mapped in 77–80% unique and 14% multimapped sites. The cut‐off values of significant change in gene expression were set as FC >2, *P*‐value <0.05. The total number of DEG and the number of upregulated/downregulated genes for both knockouts vs. either pts80 parental cell line or pts80 EV ctrl, respectively, are described in Table 8.

**Table 8:** The number of significant DEG in ALDH1A1 knockouts in comparison to pts80 parental cell line or pts80 EV ctrl was detected by RNAseq.

KO	Vs.	Genes tested	Changed	Upregulated	Downregulated	Method used
1B6 KO	Parental cell line	30 012	1	0	1	DESeq2
1B6 KO	Parental cell line	30 012	11	6	5	edgeR
1B6 KO	EV	30 012	335	208	127	DESeq2
1C3 KO	Parental cell line	30 012	20	6	14	DESeq2
1C3 KO	EV	30 012	79	46	33	DESeq2

RNA seq confirmed the significant decrease of *ALDH1A1* expression in both knockouts (FC = 0.196, *P* = 0.00327 for 1C3 KO and FC = 0.22, *P* = 1.81 × 10^−6^ for 1B6 KO). The expression profile of 1B6 KO vs. parental cell line showed only a few DEGs using the edgeR method, and just the *ALDH1A1* gene expression change was significant using the DESeq2 method. Generally, edgeR is less sensitive than DESeq2 and yields a higher number of DEGs. The expression profile of 1C3 KO was compared to the expression profile of the pts80 parental cell line. Together, 20 genes were differentially expressed; among them, six genes were upregulated, 14 genes were downregulated, and one gene was non‐coding (Fig. 7C–E). The complete list of DEG, their log2 FC, and *P*‐values are in Table S4. Among them, several genes involved in CRC carcinogenesis and/or metastatic spread were identified. Subsequently, the expression of 17 selected genes was tested by RT‐qPCR. From these, the significant upregulation in 4 genes (*TMTC1, CTH, STC1, SMOX*) and the significant downregulation in 8 genes (*TBXAS1, BCAS1, LARGE1, CYP3A5, AFAP1L2, CDH6, TNFSF15* and *PCSK9*) was confirmed by RT‐qPCR (FC > 2, *P* < 0.05) (Table 9, Fig. 7F).

**Table 9:** Selected cancer‐associated DEG detected by RNAseq and confirmed by RT‐qPCR in 1C3 knockout vs. parental pts80 counterpart. ↑—upregulation, ↓—downregulation, OS—overall survival.

Gene	Protein function	RNAseq	RT‐qPCR	CRC associated	References
*TMTC1*	O‐mannosylation of ITGβ1 & β4	15.31 × ↑	3.43 × ↑	No, but mediates cell migration and invasion in ovarian ca	[40]
*CTH, CSE*	Cysteine biosynthesis & sulfur metabolism	6.38 × ↑	2.0 × ↑	Stimulated by Wnt pathway, promotes CRC proliferation and migration *in vitro*, xenograft growth	[115]
*STC1*	Activation of ITG pathway	3.95 × ↑	3.36 × ↑	Prognostic marker of poor prognosis in refractory mCRC	[116]
*SMOX*	Produces ROS, cell proliferation, invasion	2.8 × ↑	2.25 × ↑	Prognostic marker of OS, CRC cell proliferation, invasion	[65]
*TBXAS1, CYP5A1*	Homeostasis, prostaglandin E2 synthesis	−3.1 × ↓	−2.14 × ↓	Overexpressed in CRC tumor	[117]
*BCAS1*	Oncogene	−4.18 × ↓	−2.68 × ↓	Mutated in cetuximab‐resistant CRC patients	[118]
*LARGE1*	Promotes cell survival and differentiation	−5.1 × ↓	−14.2 × ↓	Paralog of LARGE1 that was downregulated in CRC	[119]
*CYP3A5*	Drug metabolism	−5.1 × ↓	−2.2 × ↓	Associated with CRC tumor response to irinotecan therapy	[120]
*AFAP1L2*	Forms protein complexes, mediates tumor cell survival, migration	−5.24 × ↓	−4.35 × ↓	Overexpressed in CRC tumor	[121]
*CDH6*	Adhesion molecule	−5.5 × ↓	−2.13 × ↓	Promotes migration/invasion	[118]
*TNFSF15, TL1A*	TGF‐β/Smad3 signaling as mediator of TL1A‐regulated CRC cell metastasis	−6.26 × ↓	−3.03 × ↓	CRC cell growth, metastasis, inflammation, infiltration	[122]
*PCSK9, PC9*	Induces GGPP synthesis & activates KRAS/MEK/ERK signaling	−8.35 × ↓	−5.84 × ↓	Induces APC/KRAS‐mutant CRC cell growth *in vitro* and *in vivo*	[123]

Three of the upregulated genes (Table 9) in 1C3 KO, *CTH* (*CSE*, Cystathionine gamma‐lyase), *STC1* (Stanniocalcin‐1), and *SMOX* (Spermine oxidase) are associated with poor prognosis of CRC patients together with increased invasiveness, migration, and metastatic ability [86, 90, 96]. The most upregulated *TMTC1* (Transmembrane O‐mannosyltransferase targeting cadherins 1) has a role in protein–protein and protein‐lipid interactions and O‐mannosylation of ITGβ1 and ITGβ4. Till now, it has not been linked to CRC, but it also mediates cell migration and invasion in ovarian cancer [40]. The *CDH6* (Cadherin 6) is downregulated both in 1C3 KO and 1B6 KO clones. β‐catenin binds directly to the cytoplasmic tail of cadherins, leading to regulation of the actin cytoskeleton. Cells with *CDH6* downregulation are likely spindlier, fibroblast‐like, and more migratory and invasive [41]. Altogether, it could reflect the potential of 1C3 KO cells to disseminate rapidly and form distant metastases.

The most downregulated gene in 1C3 knockout was *LARGE1*, which promotes cell survival and differentiation in cancer. Fourteen times downregulated, *LARGE1* together with decreased *ALDH1A1* and subsequent decrease of retinoid acid could take part in the reduction of cell differentiation, which is visible in histological specimens of 1C3 knockout's xenografts.

ALDH1A1 directly catalyzes the conversion of retinaldehyde to retinoic acid (RA). As the consequence of attenuated ALDH1A1 expression in 1C3 and 1B6 knockouts, five RA‐responsive genes were downregulated (*CYP26A1, RARRES, RARα, FOLR1, PLAAT4*) compared to the pts80 cell line by RT‐qPCR (Fig. 7G). Other RA‐responsive genes (*LYN, RARβ, STRA6, TGM2*) were not changed significantly (FC > 2, *P* < 0.05).

The RNAseq and RT‐qPCR displayed the decreased expression of genes *TBXAS1* (*CYP5A1*, thromboxane A synthase), *CYP3A5* (belonging to Cytochrome P450, xenobiotics and drug detoxification), and *CYP26A1* (RA‐responsive gene). ALDH1A1 and CYP enzymes are involved in detoxification pathways, homeostasis, and drug metabolism.

To elucidate the increased invasive and metastatic properties of pts80 ALDH1A1 knockouts, stem cell markers and EMT genes were examined using RT‐qPCR. *Nestin, Oct4*, and *SNAI2* were upregulated, and *CEA* and *CK20* were downregulated in both knockouts (Fig. 7H). Elevated *Oct4* expression (5.3 times more than parental line in 1C3 KO, 5.1 times in 1B6 KO) could increase self‐renewal and differentiation abilities similar to CSCs. Another stem cell marker, Nestin (3.1× higher in 1C3 and 3.8× in 1B6 KO), as an intermediate filament, could increase cancer cell invasiveness by reducing cell stiffness. *SNAI2* as an EMT‐regulator increased 4.1× in 1C3 and 5.5× in 1B6 KO. Downregulation of *CEA* and *CK20* mRNA reflects reduced CRC cell differentiation. Other stem cell markers and EMT markers (EPCAM, CD133, Sox2, Twist1, VIM) were also tested, but none showed significant changes (FC >2, *P* < 0.05). The Pts80 line did not express the *MACC1* gene, and *LGR5* and CD44v6 expression are at the limit of detection.

Differentially expressed genes confirmed by RT‐qPCR detected after the CRISPR‐Cas9 editing of ALDH1A1 were analyzed by Gene Set Enrichment Analysis (GSEA) (Table S5). The analysis confirmed that genes up‐ and downregulated after *ALDH1A1* attenuation were enriched in datasets associated with vitamin D metabolism and the Wnt pathway (Fig. 7I). The retinol pathway is one of the most important among the enriched metabolic pathways (Fig. 7J).

## 4 Discussion

A newly established colorectal cell line named pts80 accurately models the characteristics of poorly differentiated colorectal adenocarcinoma with significant ALDH1A1 overexpression. Its biological, genomic, and molecular characterization contributes to the knowledge regarding CRC carcinogenesis and metastasis and, together with the clones with attenuated ALDH1A1 function, can enlighten the role of ALDH1A1 as a stem cell marker.

The establishment of patient‐derived cell lines requires cell adaptation to new environmental conditions under distinct selection pressures, and their propagation continuously selects the most viable and most rapidly proliferating cells. Moreover, tumor cells are often deficient in the ability to maintain genome integrity; their genomic instability makes them susceptible to rapid acquisition of additional genetic insults (Table 3) throughout propagation [42]. Thus, the passaging of surviving cells leads to the selection and stabilization of genetic variants over the original tumor cell populations [43]. Since CNV analysis was performed after more than 50 passages of the pts80 cells, there was a reduction of CNV counts in the genome of the derived cell line, while many chromosomes were presented in a single copy (Table 1, Fig. 2E).

According to Fearon and Vogelstein's model of CRC carcinogenesis, the alteration of normal colonic epithelial cells to polyps, adenomas, and carcinomas is linked to accumulating genetic changes [44]. An early crucial step is the disruption of the WNT signaling pathway, which occurs by the disruption of the *APC* gene or by the loss of its locus in chromosome 5q. Subsequent accumulation of mutations in *KRAS* involved in the MAPK signaling pathway, losses on the long arm of chromosome 18 affecting the TGF‐β signaling pathway, and mutations in *TP53* or loss of the short arm of chromosome 17 (17p), where *TP53* is located, result in the formation of cancer [44, 45].

In the pts80 original tumor, mutations were detected in three cancer‐associated genes: TP53 (C135Y), BRAF (G466E), and SMAD6 (Y476C), and the loss of chromosome 18 was present. This *BRAF* mutation belongs to class III mutation with low kinase activity. Mutated BRAF protein forms heterodimers with CRAF that carry oncogenic activity, and this heterodimer complex activates the MAPK–ERK pathway [46]. However, no *APC* mutation or loss of its locus was detected. Later events in CRC carcinogenesis include deletions on chromosome 18q and inactivation of the tumor suppressor gene TP53 on chromosome 17p. Loss of chromosome 18q is detected in 70% of CRCs, with many candidate tumor suppressor genes located on it: deleted in colon cancer (DCC), SMAD4 (DPC4), and SMAD2. Mutations identified in the SMAD genes are rare in CRC, but SMAD proteins regulate the TGF‐β pathway and cell cycle control [47].

Gain of chromosome 20 was detected in pts80 tumor tissue that is known to be involved in the transformation of adenomas into CRC carcinomas [48]. In addition, amplification in 20q is associated with a worse prognosis of CRC [49, 50] because the amplified genes in 20q belong to several signaling pathways that may be crucial in CRC development. During the early stage of CRC, 18q losses are present in polypoid adenomas [51], which is the case of the pts80 tumor. As a next step, gains of 8q, 13q, and 20q, and losses of 8p, 15q, 17p, and 18q are typical for the adenoma‐to‐carcinoma progression [48, 52]. Except for 17p, all chromosomal alterations are present in the pts80 tumor (Table 1, Fig. 2D). Besides these already described somatic genetic alterations, several patients' specific mutations have been detected, including genes *GRIP1* and *CTNNBL1* that could trigger the WNT pathway, *SMAD6* mutation affecting the TGF‐β pathway, and *URGCP* mutation with potential consequences in NF‐κB signaling that represent key mechanisms in CRC development. Other mutations detected in *LZTS3* and *PDCD4* tumor suppressor genes, as well as *CDH8, PCDH17*, and *NRXN2* adhesion molecules, could represent individual, patient‐specific mutations or passenger mutations (Table 2). The patient's tumor tissue was frozen after surgery and stored in the tissue biobank and then used for gDNA and RNA isolation for WES and RNA sequencing. We have to assume the large heterogeneity of the populations within the patient's tumor mass, together with the impact of the tumor microenvironment on the RNA expression profile.

We observed extensive gains and losses of genetic material in the tumor tissue of pts80 patient (Fig. 2D). Such chromosome instability is known to occur in 80% of tumors [53] and may promote carcinogenesis and tumor evolution by increasing genetic heterogeneity [54]. Thus, the tumor represents a heterogeneous mass of different cell types, and genetic changes in the malignant cell population are considered a process of diversification that leads to the survival of the fittest clones [55]. Such alterations occurred during the establishment of the pts80 cell line and were shown to have an impact on the functional and morphological properties of cells. The functional traits of the original tumor have shown a higher representation of glandular cells with similar oval morphology and more interstitial supportive tissue inside the tumor mass (Table 5), suggesting more differentiated adenocarcinoma. On the other hand, pts80 subcutaneous xenografts have shown the presence of reticular and tubular cells of different abnormal shapes with almost no support of interstitial tissue, and together with increased vimentin production, indicating the non‐differentiated, more aggressive adenocarcinoma.

The *in vitro* cultivation of the cell population derived from the patient's tumor tissue continued with the accumulation of additional mutations in the cell line. Detailed analysis was performed after passage 140 at a stabilized cell line. WES and RNAseq revealed distinct mutations and different DEGs compared to the pts80 bulk tumor. Some alterations may be attributed to cell stabilization during passaging and to selection pressure, as clones that can proliferate *in vitro* gain an advantage. In the pts80 cell line, there are 17 CRC‐associated mutated genes identified among 37 likely pathogenic mutations (Table 3), which were used for enrichment analysis based on the Combined Pathogenicity Score by the KEGG pathway database (Table S2, Fig. 2G). Their potential impact is pleiotropic and interferes with multiple essential pathways regulating cellular processes, dysregulation of which leads to cancer progression. The most influential are metabolic and cAMP signaling pathways, suggesting alterations in energy metabolism and cellular signaling; calcium signaling implicates changes in cell motility and apoptosis. Pathways in cancer (particularly PI3K‐Akt, Ras, NFκB, JAK–STAT, and Hedgehog pathways) and neuroactive ligand‐receptor interaction were also identified as enriched, indicating broad impacts on cell proliferation and survival.

Pts80 cell line is unique by significant overexpression of the *ALDH1A1* gene (located in chromosome 9q21.13). It participates in the cellular functions essential for cell survival along with cell protection mechanisms. It irreversibly oxidizes endogenous and exogenous aldehydes to corresponding acids in the presence of NAD+, including the conversion of retinal to retinoic acid, which modulates cell proliferation and differentiation [4].

Usually, high *ALDH1A1* expression correlates with poor prognosis, tumor cell proliferation, treatment resistance, and tumorigenicity. The clinical significance of ALDH1A1 expression in CRC is controversial among published studies. High ALDH1A1 expression was associated with lymph node metastasis [56], right‐sided tumors, lymphovascular invasion, and perineural invasion [57]. Wang et al. divided ALDH1A1 expression according to localization to cytoplasmic and nuclear. They stated that cytoplasmic expression is not a suitable prognostic marker of CRC. They found that ALDH1A1 nuclear expression level in low‐grade adenomas was predominantly higher than those in high‐grade adenomas, primary cancer, and liver metastases, concluding ALDH1A1 nuclear expression is associated with a better outcome [58]. Similarly, ALDH1A1 overexpression in SW480 cells decreased cell proliferation and invasiveness *in vitro* but increased tumorigenicity. They concluded that ALDH1A1 is differentially overexpressed in CRC liver metastases and may play a dual role, functioning as both tumor suppressors and metastasis promoters in CRC [59]. Therefore Yue et al. [60], in a systematic review, stated that ALDH1A1 acts bidirectionally and cannot be considered a prognostic factor for CRC. Our results concerning increased invasivity and metastatic potential after genetic editing of ALDH1A1 in pts80 and HT‐29 cell lines are in concordance with recent papers.

Consistently, CNV analysis of the original tumor tissue has shown nearly whole chromosome 9 duplication, thus increasing the ALDH1A1 gene dosage and potentially promoting malignant phenotype. However, after pts80 cell line stabilization, chromosome 9 bearing the *ALDH1A1* gene was present in two copies, and ALDH1A1 was not affected by any structural variant (within the limit of detection, i.e., longer than 500 kb). Transcriptome profile and pathway enrichment analysis in the pts80 cell line showed activation of oxidative stress response, which could be linked to *ALDH1A1* overexpression.

Using CRISPR‐Cas9 genome editing, we achieved about 13–15‐fold decrease in *ALDH1A1* mRNA expression (Fig. 3F) and significant reduction of ALDH activity in 1C3 and 1B6 KO mutants (Fig. 3D). The role of ALDH1A1 isoform can be partially replaced by ALDH1A3 or ALDH1A2 enzymes due to genetic compensation [61]. We tested the *ALDH1A2*, *ALDH1B1*, *ALDH2*, *ALDH3A1* (Table 6), and *ALDH1A3* (Fig. 3F) mRNA expression levels in 1C3 and 1B6 KOs, but no significant changes were detected.

Two *in vitro assays* based on single‐cell‐derived colony formation confirmed that both the pts80 and HT‐29 cell lines (and the transduction controls pts80 EV and HT‐29 EV) can grow in non‐adherent conditions and proliferate from a single active cell into large colonies. ALDH1A1 editing results in a reduction in the number of created colonies, but also in their size. Luminescent evaluation of viable cells at the end point of the colonsphere‐forming assay confirmed a decrease in viable cells in ALDH1A1 knockouts. The reduced ability to form large colonies (decreased proliferation rate) in soft agar in knockouts reflects reduced growth after s.c. administration on immunodeficient mice (Fig. 5A,H). As a next step, an *in vivo* extreme limiting dilution assay was performed to calculate the stem cell frequency for the pts80 parental line and HT‐29, and for one representative knockout, to reduce the number of animals in accordance with the 3Rs. HT‐29 grew more rapidly at higher concentrations, leaving G5‐3 KO behind by 10–14 days. At high concentrations (10^6^–10^4^), 1C3 KO tumors appeared several days after parental pts80, but the switch occurred at the lowest concentration, when one hundred injected 1C3 KO cells displayed aggressive traits, giving rise to five tumors from eight applications. This fact refers to the acquisition of aggressive properties also associated with metastatic ability and will be the subject of further study. Aggressive behavior of 1C3 KO is supported by the rise of mRNA expression of stem cell markers Oct4 and Nestin, and EMT marker SNAI2. Loss of differentiation was confirmed by histological examination of the 1C3 and 1B6 KO xenografts, but also by the decrease of CEA and CK20 in their cell lines (RT‐qPCR) and in their xenografts (immunohistochemistry).

Migration assay reported significantly increased invasive properties of 1C3 and 1B6 KOs compared to the parental cell line, which is in concordance with the increased ability to disseminate and form distant metastases observed *in vivo*. The ALDH1A1 attenuation resulted in extensive metastatic spread of cancer cells, affecting multiple organs in experimental animals. The involvement of ALDH1A1 as a cancer stem cell marker important for tumorigenicity is well described [62], but its suppression has not yet been associated with metastatic potential in CRC. Retinoic acid triggers cell differentiation and ALDH1A1 genetic attenuation, followed by CYP26A1, RARRES, RARα, FOLR1, and PLAAT4 downregulation, resulting in the growth of non‐differentiated xenografts with a more aggressive phenotype. Folate receptor alpha (FOLR1) is often overexpressed on the cell surface of cancer cells, and it is also associated with aggressive tumor behavior and worse survival in rectal cancer [63]. The FOLR1 is often used as a target molecule in nanoparticle‐mediated cancer therapy. Downregulation in 1B6 and 1C3 KO cells could eliminate this potential target.

Differentially expressed genes between 1C3 KO and pts80 parental cell line (Table 7) displayed decreased expression of genes linked to cell–cell adhesion (Cadherin 6, LARGE1) and cytoskeleton rearrangement (AFAP1L2 or XB130) [64] participating in cell motility and invasion. The STC1 gene involved in angiogenesis is upregulated, potentially promoting the new vessel formation. The BCAS1 protein enhancing cell proliferation is downregulated in 1C3 KO cells, which is in concordance with a decreased level of Ki67 proliferation marker in 1C3 KO xenografts. It was reported that SMOX downregulation decreased metastatic capacity in CRC [65], but oppositely, SMOX was upregulated in the 1C3 clone. The role of TBXAS1 in cancer is not fully explained, but it is involved in the synthesis of thromboxane A2, a potent inducer of platelet aggregation. Platelet interaction with tumor cells leads to their activation and promotion of tumor progression and metastasis [66]. The platelets' rise aggravated CRC development, while inhibition of platelet adhesion reduced tumor growth [67]. Alterations in ALDH1A1 expression can disrupt the balance of metabolic intermediates, affecting the expression of CYP enzymes TBXAS1 (CYP5A1), CYP3A5, and CYP26A1.

The previous study of shRNA‐linked suppression of overexpressed ALDH1A1 in a human colon cancer cell line COLO320 analyzed subsequent transcriptomic, proteomic, and untargeted metabolomic changes [68]. The combined analysis identified altered pathways related to lipid metabolism (such as omega‐3 and omega‐6 fatty acid metabolism, *de novo* fatty acid biosynthesis, and glycophospholipid biosynthesis), as well as energy metabolism (including pyrimidine metabolism, the TCA cycle, purine metabolism, and di‐unsaturated fatty acid beta‐oxidation). Both *WNT10A* and *c‐MYC* were significantly downregulated in the COLO320 cell line with attenuated ALDH1A1 [68]. We also observed changes in the transcriptome profile of the 1C3 KO clone affecting the Wnt pathway (Fig. 7I).

We have established a novel epithelial cell line from rectal adenocarcinoma and characterized its biological properties *in vitro* and *in vivo*. Due to the significantly increased intrinsic ALDH1A1 overexpression, the pts80 cell line can serve as a model for studying the role of ALDH1A1 in stemness, carcinogenesis, and metastasis. CRISPR‐Cas9 genome editing has been previously used in colorectal cancer research in adherent cell lines, spheroids, and organoids [9], but never for genetic attenuation of ALDH1A1, followed by genomic, transcriptomic, and functional analysis as follows:

As we have shown, structural variants (gains of 8q, 13q, and 20q, and losses of 8p, 15q, and 18q) and somatic mutations (in *TP53*, *BRAF, SMAD6*) detected by WES in the patient's rectal adenocarcinoma tissue are typical for the adenoma‐to‐carcinoma progression via the proposed model of CRC carcinogenesis. The attenuation of ALDH1A1 led to increased invasive capacity and metastatic potential. Twelve differentially expressed genes were identified and confirmed in the knockouts, indicating the inhibition of proliferation activity and the promotion of invasive properties. Our results could expand the knowledge about carcinogenesis and metastatic dissemination of CRC. The identified interconnections could lead to the revealing of novel therapeutic targets or biomarkers for colorectal cancer.

## 5 Conclusions

We have established a novel epithelial cell line from rectal adenocarcinoma. For significant ALDH1A1 overexpression, the pts80 cell line can serve as a model for studying the role of ALDH1A1 in stemness, carcinogenesis, and metastasis. The attenuation of ALDH1A1 led to increased invasive capacity and metastatic potential. Twelve differentially expressed genes were detected and confirmed in pts80 knockouts, confirming the inhibition of proliferation activity and promoting invasive properties.

## Conflict of interest

All authors declare no conflict of interest.

## Author contributions

MP: Conceptualization, Data curation, Methodology, Investigation, Validation, Visualization, Writing—Original draft preparation and revision, Funding acquisition. ZK: Conceptualization, Methodology, Investigation, Writing—Original draft preparation, Funding acquisition. OP: Validation, Visualization, Writing—Original draft preparation and revision. KP: Investigation. SG: Methodology and Investigation. PM: Methodology and Investigation. KJ: Investigation. MB: Methodology, Investigation, Data processing. ES: Investigation. BS: Investigation. ST: Methodology, Investigation, Visualization. VB: Data curation, Software. NB: Validation, Data curation, Software. BT: Conceptualization, Methodology, Investigation, Funding acquisition. MH: Data curation, Software, Formal analysis. JB: Data curation, Software, Formal analysis. MT: Investigation. PD: Investigation. GK: Methodology, Investigation, Validation. VR: Investigation. NM: Investigation. EZ: Investigation. DP: Investigation. MMe: Investigation. TS: Funding acquisition, Project administration, Supervision. MMa: Conceptualization, Methodology, Investigation, Validation, Data curation, Writing ‐ Original draft preparation and revision, Funding acquisition, Project administration. All authors had access to the data of this study and had reviewed and approved the final manuscript.

## Ethics approval and consent to participate

The tissue and blood of the patient were collected under written informed consent, and the study was approved by the Ethics Committee of the National Cancer Institute in Bratislava (study GIT 01). The animal studies were evaluated by the Ethics Committee of the Biomedical Research Center of SAS and were approved by the national competence authority – State Veterinary and Food Administration of the Slovak Republic (Ro‐207/18‐221/3, Ro‐290‐3/2020‐220).

## Supporting information


**Table S1.** Primers and probes used for expression analysis and detection of human gDNA in mouse tissue by duplex PCR.

**Table S2.** WES‐based enriched pathways based on somatic mutations in pts80 cell line.

**Table S3.** The list of differentially expressed genes and dysregulated signaling pathways in the pts80 cell line compared to the patient's tumor tissue.

**Table S4.** The list of differentially expressed genes in clone 1C3 with attenuated ALDH1A1 expression compared to parental pts80 cell line.

**Table S5.** Enrichment analysis GSEA Canonical pathways between 1C3 vs. parental.

## Data Availability

Data generated and analyzed during this study are included in this published article and supplementary files or are available from the corresponding author upon request.
